# A comparison of postnatal arterial patterns in a growth series of giraffe (Artiodactyla: *Giraffa camelopardalis*)

**DOI:** 10.7717/peerj.1696

**Published:** 2016-02-16

**Authors:** Haley D. O’Brien, Paul M. Gignac, Tobin L. Hieronymus, Lawrence M. Witmer

**Affiliations:** 1 Biological Sciences, Ohio University, Athens, OH, United States; 2 Department of Anatomy and Cell Biology, Oklahoma State University Center for Health Sciences, Tulsa, OK, United States; 3 Department of Anatomy and Neurobiology, Northeast Ohio Medical University, Rootstown, OH, United States; 4 Department of Biomedical Sciences, Ohio University Heritage College of Osteopathic Medicine, Athens, OH, United States; 5 Current affiliation: Department of Anatomy and Cell Biology, Oklahoma State University Center for Health Sciences, Tulsa, OK, United States

**Keywords:** *Giraffa camelopardalis*, Arterial development, Ontogeny, Anatomical imaging, CT scan, Artiodactyla, Ruminant, Carotid rete

## Abstract

Nearly all living artiodactyls (even-toed ungulates) possess a derived cranial arterial pattern that is highly distinctive from most other mammals. Foremost among a suite of atypical arterial configurations is the functional and anatomical replacement of the internal carotid artery with an extensive, subdural arterial meshwork called the carotid rete. This interdigitating network branches from the maxillary artery and is housed within the cavernous venous sinus. As the cavernous sinus receives cooled blood draining from the nasal mucosa, heat rapidly dissipates across the high surface area of the rete to be carried away from the brain by the venous system. This combination yields one of the most effective mechanisms of selective brain cooling. Although arterial development begins from the same embryonic scaffolding typical of mammals, possession of a rete is typically accompanied by obliteration of the internal carotid artery. Among taxa with available ontogenetic data, the point at which the internal carotid obliterates is variable throughout development. In small-bodied artiodactyls, the internal carotid typically obliterates prior to parturition, but in larger species, the vessel may remain patent for several years. In this study, we use digital anatomical data collection methods to describe the cranial arterial patterns for a growth series of giraffe (*Giraffa camelopardalis*), from parturition to senescence. Giraffes, in particular, have unique cardiovascular demands and adaptations owing to their exceptional body form and may not adhere to previously documented stages of cranial arterial development. We find the carotid arterial system to be conserved between developmental stages and that obliteration of the giraffe internal carotid artery occurs prior to parturition.

## Introduction

The mammalian order Artiodactyla is the most speciose clade of ungulates. According to recent estimates, artiodactyls comprise more than 220 of the 237 species of extant ungulates. Geographically, even-toed ungulates can be found on all continents (except for Antarctica) and are known to inhabit extreme environments, such as tundra, desert, and high altitude. These animals are well adapted to their herbivorous and frequently migratory life histories and have a suite of physiological specializations that are unique to them among ungulates. Beyond the four-chambered stomach characteristic of non-suid artiodactyls, nearly every living species possesses a pattern of cranial vasculature involved with sophisticated cranial thermoregulation. With the exception of the tropical mouse deer (family Tragulidae; Fukuta et al., 2007; O’Brien, 2015), adults of all species possess a subdural carotid arterial rete that supplies the majority of oxygenated blood bound for the brain (Daniel, Dawes & Prichard, 1953; Nickel & Schwarz, 1963; Kanan, 1970; Gillilan, 1974; Carlton & McKean, 1977; Frąckowiak et al., 2015). This rete is housed within the cavernous venous sinus, which receives venous blood that has been evaporatively cooled by the maxilloturbinates. Due to the high surface area of the arterial meshwork, heat rapidly dissipates across the arterial walls and into the cool venous sinus. Warmer blood is then returned to the body as cooler blood ascends to the brain. At the same time, mature artiodactyls possess either a reduced internal carotid artery (ICA) or more commonly have an occluded, fibrous remnant of extracranial (proximal) segment of this vessel (Tandler, 1899; Tandler, 1906; Van Kampen, 1905; Daniel, Dawes & Prichard, 1953; Baldwin, 1964; Gillilan, 1974; Bamel, Dhingra & Sharma, 1975; Wible, 1984; Fukuta et al., 2007).

Although the cranial arteries of mature artiodactyls are substantially different from the typical mammalian pattern, arterial development begins from the same embryonic scaffolding. All artiodactyl taxa for which embryonic data are available initially develop a homologous ICA from the 3rd aortic arch. With the exception of tragulids, the ICA diminishes in diameter or obliterates at a variable point during ontogeny. Some taxa lose a patent ICA in prenatal ontogenetic stages, including the domestic pig (*Sus scrofa scrofa*; Tandler, 1906; Von Hofmann, 1914; Llorca, 1933; Daniel, Dawes & Prichard, 1953; Wible, 1984). Other species, such as the domestic goat (*Capra hircus hircus*) and sheep (*Ovis aries*), maintain a patent ICA until shortly after parturition (Daniel, Dawes & Prichard, 1953). Finally, a patent ICA can persist for several years in large bovids (*Bos taurus*; Daniel, Dawes & Prichard, 1953; Gillilan, 1974; and *Bubalus bubalis*; Bamel, Dhingra & Sharma, 1975), and a small remnant of the ICA may persist until senescence in the dromedary camel (*Camelus dromedarius*; Lesbre, 1903; Tayeb, 1951; Kanan, 1970; Wible, 1984). The developmental mechanism driving the elimination of the ICA is currently unknown; however, the emergent pattern from the few taxa documented suggests that a functional ICA is retained further into life among artiodactyls that achieve larger adult body size and/or have longer gestation periods.

Among artiodactyls, few species face cardiovascular demands as unique as those of giraffes (*Giraffa camelopardalis*). Giraffes have many cardiovascular adaptations, largely owing to their exceptional body form—their large bodies (average: 1191.8 kg for males, 828.4 kg for females; Skinner & Chimimba, 2005) are accompanied by disproportionately long limbs and the longest neck known for any extant mammal. Major physiological challenges accompanying this unusual anatomy include dependent edema in the appendages and maintenance of cerebral blood pressure. Venous pooling in the legs is prevented by exceptional venous return mechanisms and tight, non-elastic skin (Hargens et al., 1987; Hargens et al., 1988; Mitchell & Hattingh, 1993). With a head that is an average of 2 m above the heart, blood must travel a substantial distance while fighting gravity to reach and perfuse the head. Giraffes accomplish this through a unique combination of cardiac and craniocervical vascular specializations. Blood delivery to the head depends on the magnitude of mean arterial pressure (Goetz, 1955; Goetz & Budtz-Olsen, 1955; Patterson et al., 1965; Mitchell et al., 2006; Mitchell & Skinner, 2009), largely generated by powerful, hypertrophied ventricular and interventricular walls (Mitchell & Skinner, 2009) and high arterial pressure maintained in the common carotid arteries (Goetz, 1955; Goetz & Keen, 1957; Keen & Goetz, 1957; Hargens et al., 1988; Mitchell et al., 2006). Once blood reaches the head, cerebral blood pressure may be regulated by a number of morphological and physiological specializations. Potential mechanisms include carotid-vertebral arterial anastomoses (Lawrence & Rewell, 1948) and, less likely, the carotid rete (Ask-Upmark, 1935; Goetz, 1955; Van Citters et al., 1969; Edelman et al., 1972; Mitchell, Bobbitt & Devries, 2008; O’Brien & Bourke, 2015). Following blood flow through the rete, increased flow of venous blood into non-collapsible vertebral veins may also contribute to maintaining cerebro-cervical arterial blood pressure (Hicks & Munis, 2005; Mitchell, Bobbitt & Devries, 2008).

Although cranial vasculature of adult giraffes have been well studied (see e.g., Lawrence & Rewell, 1948; Goetz & Keen, 1957; Kimani, 1987; Kimani & Opole, 1991; Ganey, Ogden & Olsen, 1990; Frąckowiak & Jakubowski, 2008; Mitchell, Van Sittert & Skinner, 2009; Davis, Brakora & Lee, 2011), growth and development have only been documented for the heart (Mitchell & Skinner, 2009). The ontogeny of giraffe cranial arteries remains undescribed but is potentially important from at least four perspectives. First, it is known that adult giraffes do not possess a fully-formed internal carotid artery (Lawrence & Rewell, 1948; Goetz & Keen, 1957; Kimani, 1987; Kimani & Opole, 1991; Ganey, Ogden & Olsen, 1990; Davis, Brakora & Lee, 2011), but it is unknown when this vessel obliterates during ontogeny. Second, the more-than-450-day gestation period of giraffes is exceptionally long when compared to other large artiodactyls (Skinner & Hall-Martin, 1975; for perspective, the gestation period of giraffes is approximately the same as that of sperm whales (Best, Canham & Macleod, 1984)). Third, giraffes are among the most massive artiodactyls and are the tallest terrestrial vertebrate alive today. Thus, both gestation and size may have important impacts on the onset of ICA obliteration. Fourth, cardiovascular function and performance are also size-dependent: blood pressure increases with strong positive allometry, which is critical because giraffes are not born with sufficiently high blood pressure to supply their heads when they are adults (Keen & Goetz, 1957; Hargens et al., 1988; Mitchell & Skinner, 2009). Here, we describe and compare the cranial arteries of neonate (stillborn) and juvenile (6 month-old) giraffes with the arterial pattern of a senescent giraffe. This is the first reported growth series of giraffe cranial arteries and the first description of giraffe arteries using precise, three-dimensional anatomical data collection methods.

## Materials

Three cadaveric giraffe specimens were utilized in this study. All specimens were obtained as loans from zoological parks and their partner academic affiliations or were donated to the Ohio University Vertebrate Collection (OUVC). No animals were sacrificed for the purpose of this study, and all required permits and permissions pertain to the transfer of specimens from zoological parks. All specimens are unrelated female Maasai giraffes (*Giraffa camelopardalis camelopardalis*). A full-term, neonate giraffe (referred to herein as “neonate”) was generously loaned by the Tulsa Zoo (Tulsa, Oklahoma, USA) to the Oklahoma State University Center for Health Sciences (ISIS no. 17249). This specimen was sectioned at the level of the occipital condyles. As a stillborn zoological specimen with no *a priori* knowledge regarding cause of death, this specimen was significantly necropsied by Tulsa Zoo staff veterinarians. This necropsy resulted in the removal of the tongue, sublingual glands, a majority of the parotid gland, and the floor of the oral cavity (including the contents of the submandibular and submental triangles), as well as complete, bilateral transection of the buccal region. Because of this extensive manipulation, arterial injection of this specimen deviated slightly from standard protocol (see below). At the time of publication, cause of death remains unknown. The second specimen is a 6-month-old (herein: “juvenile”) giraffe obtained from the Oklahoma City Zoo and accessioned at the Sam Noble Museum of Natural History (Norman, Oklahoma, USA; Specimen Number: OMNH 64236). The juvenile giraffe died during surgery to correct a major congenital defect not involving the cephalic vasculature. This specimen was sectioned at the level of the fourth cervical vertebra, enabling the arteries of the neck to be studied. The third specimen is a senescent adult giraffe donated to Ohio University by the International Center for the Preservation of Wild Animals, Inc. (The Wilds, Cumberland, Ohio, USA), and accessioned as OUVC 10513. This specimen was sectioned at the level of the atlas. All individuals described herein died of natural causes, were not fixed in alcohol or formalin, and were stored frozen until the time of study.

## Methods

Data collection followed the protocols of Holliday et al. (2006), wherein specimens are injected with a radiopaque injection medium, CT-scanned, and then rendered in three dimensions, allowing for soft- and hard-tissue interactions to be examined freely, without destructive sampling. Because all specimens were acquired at different dates and in different locations, vascular injection protocol differed for each. Ultimately, there was no significant difference in the quality of each injection. The protocols only differ with respect to circumstances of the individual specimen or locations at which the injections and scans were conducted. Injection and scanning protocols are described separately for each specimen.

### Neonate giraffe injection and scanning methods

The extensive necropsy performed on the neonate specimen necessitated that injection proceeded in multiple stages. Initially, the left common carotid artery was cannulated with 1/8-inch external diameter PVC tubing (Thermo Scientific, Waltham, MA, USA). Tubing was fixed in place with surgical ligature, adhesive, and hemostats. The arterial system was then manually flushed with warm water for 10 min, followed by perfusion with 90 mL of 10% One-Point anticoagulant solution. Prior to flushing the neonate specimen, steps were taken to mitigate latex extravasation through the extensive necropsy incisions. Large severed vessels were clamped with hemostats and the oral cavity was filled with cotton balls that were soaked in glacial acetic acid (10% CH_3_COOH). Acetic acid immediately sets latex, preventing unwanted flow of the injection medium from the many oral vessels damaged during necropsy. Incisions were made in midline structures (i.e., mandibular and maxillary labia, dorsal nasal region, medial base of ossicones) to monitor for full perfusion of major vessels. Following initial preparation, the specimen was manually injected with a solution of 40% Liquid Polibar Plus barium sulfate suspension (BaSO_4_; E-Z-Em, Westbury, NY, USA) in 60% red liquid latex injection medium (Ward’s, Rochester, NY, USA). Initial injection continued until latex extravasated through the contralateral common carotid artery (O’Brien & Williams, 2014). Due to the prevalence of transected arteries in the facial region, an attempt was made to locate arteries along these cut borders. When candidate vessels were located, these were individually perfused. Subsequent injections were performed using the same injection medium, but smaller catheters (18-gauge angiocatheters (Becton Dickinson, Franklin Lakes, NJ, USA)). This resulted in the injection of a large facial vein, which was not reconstructed in the 3-D models. The total volume of injected medium was 35mL. Throughout the latex injection process, small vascular leaks were sealed using glacial acetic acid.

The neonate specimen was CT-scanned at the Oklahoma State University School of Veterinary Medicine, using a GE LightSpeed QX/i 4-slice CT scanner at 0.625 mm slice thickness, 100 kVp, and 345 mA, yielding 585 axial slices. This resulted in an initial voxel size of 0.436 × 0.436 × 0.625 mm. The data was imported into Avizo 7.0 (Visualization Sciences Group), and up-sampled to 0.1 × 0.1 × 0.1 mm.

### Juvenile giraffe injection and scanning methods

The left common carotid artery was cannulated with 1/8-inch PVC tubing (Thermo Scientific, Waltham, MA, USA). Tubing was fixed in place with surgical ligature and adhesive. The arterial system was then manually flushed with warm water for 10 min, followed by a flush of 600 mL of 10% One-Point anticoagulant solution. After preparation, the specimen was manually injected with a solution of 40% Liquid Polibar Plus barium sulfate suspension in 60% red liquid latex injection medium. Perfusion continued until latex emerged through the contralateral common carotid artery, following the perfusion criteria of O’Brien & Williams (2014). The volume of radiopaque latex injected was approximately 150 mL. Acetic acid (10% glacial acetic acid solution) was used through the duration of the injection to set any extravasated latex.

The juvenile specimen was CT-scanned at the Oklahoma State University School of Veterinary Medicine, using a GE LightSpeed QX/i 4-slice CT scanner, at 0.625 mm slice thickness, 140 kVp, and 165 mA, yielding 965 axial slices. This resulted in an initial voxel size of 0.752 × 0.752 × 0.625 mm. The scan data was then imported into Avizo 7.0 and up-sampled to 0.25 × 0.25 × 0.25 mm.

### Adult giraffe injection and scanning methods

The injection of the adult specimen (OUVC-10513) was performed as a differential-contrast, dual-vascular injection whereby the carotid arteries and jugular veins were cannulated and separately injected with contrast media of differing densities (Holliday et al., 2006). The facial veins were also cannulated rostral to the orbit, and venous injection medium was injected in each direction. The contrast medium consisted of a mixture of latex injection solution and Liquid Polibar Plus barium sulfate suspension. Arteries and veins were injected with a 40% and 20% barium solution, respectively. Arteries were given a higher concentration of barium because their lumina tend to be smaller than those of veins, allowing small vessels to be better visualized. The more capacious veins could be visualized with the lower-density medium simply because of their larger volume. The differential barium concentration with the arteries and veins produced correspondingly different brightness levels in the CT data, allowing the arteries to be segmented separately from the veins. As the focus of our research involves the arterial system, the venous system is not described herein.

The adult specimen was then CT scanned at OhioHealth Hospital in Athens, Ohio, using a Toshiba Aquilion 64 CT scanner at 0.5 mm slice thickness, 120 kVp, and 400 mA, yielding 2480 axial slices. This resulted in an initial voxel size of 1.03 × 1.03 × 0.3 mm. The scan data were then imported into Avizo 7.0, and re-sampled to 0.6 × 0.6 × 0.6 mm.

### Digital data processing

All scan data were up-sampled to improve resolution in Avizo. Up-sampling decreases the average voxel size without affecting the inherent quality of the data. This is a reliable technique for generating a visually smoother surface upon reconstruction. Because a 40% barium-latex solution yields stark contrast between the arteries and the surrounding hard tissues, the skull and arteries were segmented based on distinctive gray-scale values. Manual segmentation was then employed to verify and edit the accuracy of the model. Segmented morphology was subsequently rendered in three dimensions with minimal use of smoothing algorithms (i.e., “Unconstrained Smoothing” setting of 1.7 (from a scale of 0 to 9)). Vascular nomenclature largely follows that codified in the Nomina Anatomica Veterinaria (2012), with accepted terminology in Latin following the first reference to a vessel.

## Results and Discussion

### Cranial arterial patterns in the adult giraffe

**Figure 1:**
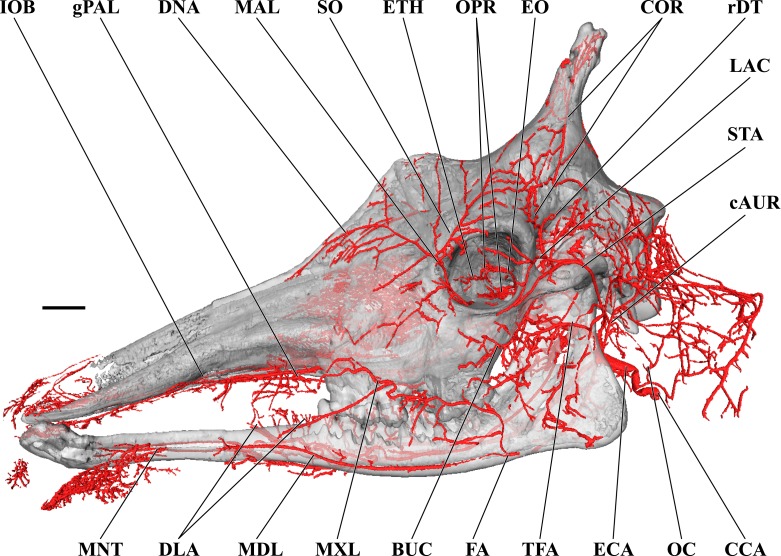
Cranial arteries of the adult giraffe viewed from the lateral perspective. For clarity, smaller branches are unlabeled. These are referenced in Table S1. *Abbreviations*: BUC, Buccal Artery; cAUR, Caudal Auricular Artery; CCA, Common Carotid Artery; COR, Cornual (ossicone) Artery; DLA, Deep Lingual Artery; DNA, Dorsal Nasal Artery; ECA, External Carotid Artery; EO, External Ophthalmic Artery; ETH, Ethmoidal Arteries; FA, Facial Artery; gPAL, Greater Palatine Artery; IOB, Infraorbital Artery; LAC, Lacrimal Artery; MAL, Malar Artery; MDL, Mandibular Labial Artery; MNT, Mental Artery; MXL, Maxillary Labial Artery; OC, Occipital Artery; OPR, Ophthalmic Retia; rDT, Rostral Deep Temporal Artery; SO, Supraorbital Arteries; STA, Superficial Temporal Artery; TFA, Transverse Facial Artery. Scale bar is 5 cm.

**Figure 2:**
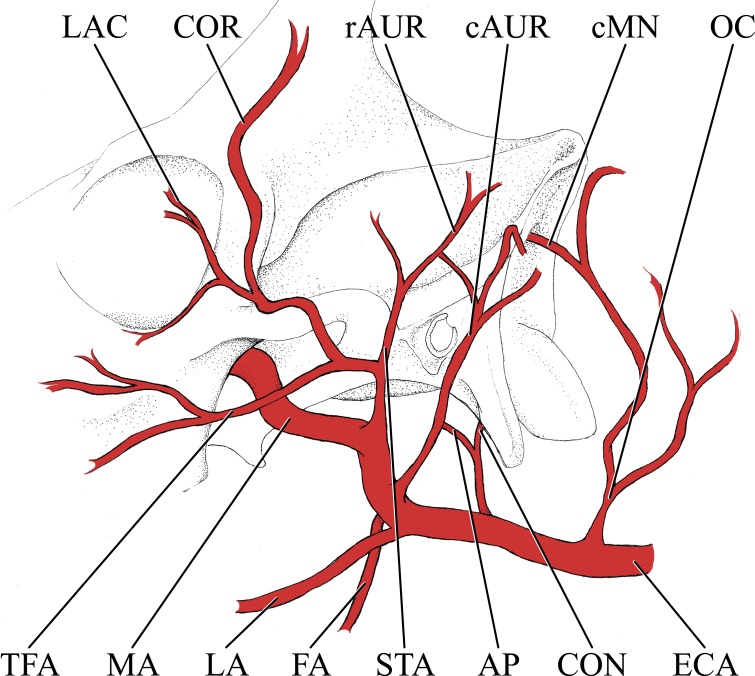
Simplified illustration of the major branches of the external carotid artery of the giraffe. *Abbreviations*: AP, Ascending Pharyngeal Artery; cAUR, Caudal Auricular Artery; cMN, Caudal Meningeal Artery; CON, Condylar Artery; COR, Cornual Artery; ECA, External Carotid Artery; FA, Facial Artery; LA, Lingual Artery; LAC, Lacrymal Artery; MA, Maxillary Artery; OC, Occipital Artery; rAUR, Rostral Auricular Artery; STA, Superficial Temporal Artery; TFA, Transverse Facial Artery.

**Figure 3:**
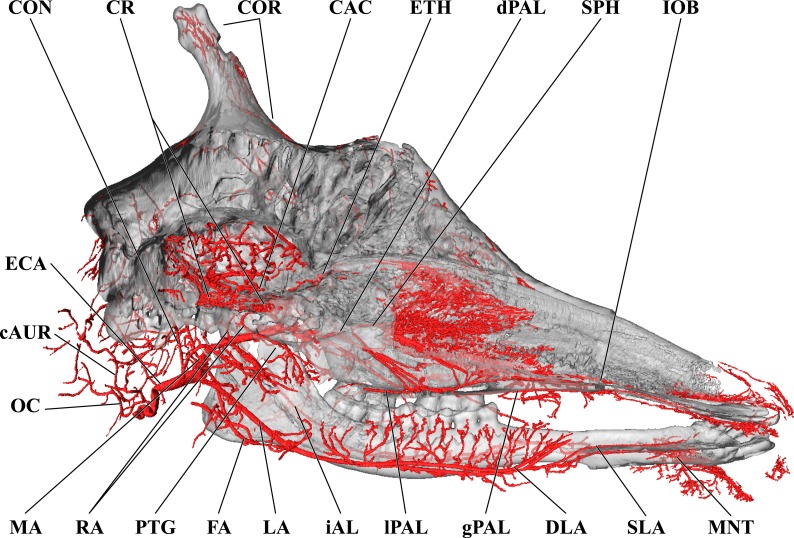
Cranial arteries of the adult giraffe, medial perspective. Cranial arteries of the adult giraffe viewed from a midline sagittal section. For clarity, smaller branches are unlabeled. These are referenced in Table S1. *Abbreviations*: AP, Ascending Pharyngeal Artery; CAC, Cerebral Arterial Circle (of Willis); CON, Condylar Artery; COR, Cornual Artery; CR, Carotid Rete; DLA, Deep Lingual Artery; dPAL, Descending Palatine Artery; ECA, External Carotid Artery; FA, Facial Artery; gPAL, Greater Palatine Artery; iAL, Inferior Alveolar; IOB, Infraorbital Artery; LA, Lingual Artery; lPAL, Lesser Palatine Artery; MA, Maxillary Artery; MNT, Mental; OC, Occipital Artery; PTG, Pterygoid Arterial Branches; RA, Ramus Anastomoticus; SLA, Sublingual Artery; SPH, Sphenopalatine Artery. Scale bar is 5 cm.

**Figure 4:**
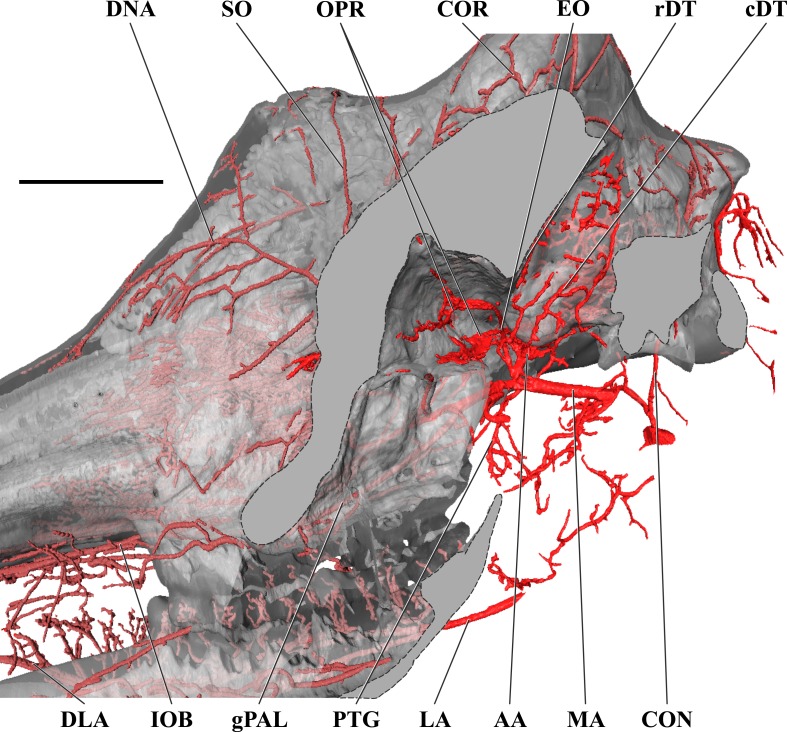
Orbital arteries and branches of the maxillary artery of the adult giraffe. Sagittal section of the adult giraffe cranium, highlighting the orbital arteries and branches of the maxillary artery that are deep to the mandibular ramus. Grey blocks indicate plane of skull bone sections (clockwise from top): maxillary and frontal bones, squamous temporal/zygomatic arch; occipital condyle; mandible. For clarity, smaller branches are unlabeled. These are referenced in Table S1. *Abbreviations*: AA, Arteria Anastomotica; cDT, Caudal Deep Temporal; CON, Condylar Artery; COR, Cornual Artery; DLA, Deep Lingual Artery; DNA, Dorsal Nasal Artery; EO, External Ophthalmic; gPAL, Greater Palatine Artery; IOB, Infraorbital Artery; LA, Lingual Artery; MA, Maxillary Artery; OPR, Ophthalmic Rete; PTG, Pterygoid Arterial Branches; RA, Ramus Anastomoticus; rDT, Rostral Deep Temporal; SO, Supraorbital Artery. Scale bar is 5 cm.

**Figure 5:**
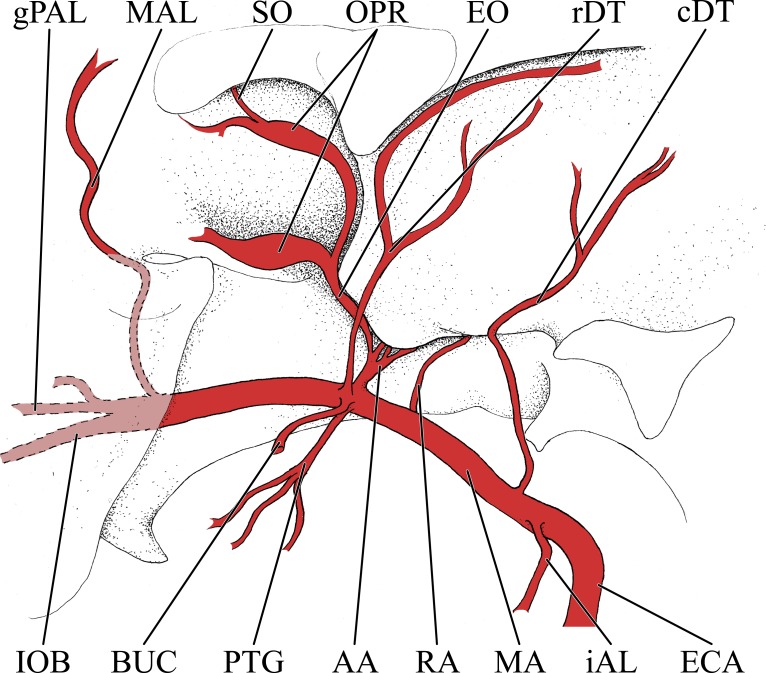
Simplified illustration of the major branches of the maxillary artery of the giraffe. Zygomatic arch and lateral extent of orbital bones are not pictured. *Abbreviations*: AA, Arteria Anastomotica; BUC, Buccal Artery; cDT, Caudal Deep Temporal Artery; ECA, External Carotid Artery; EO, External Ophthalmic Artery; gPAL, Greater Palatine Artery; iAL, Inferior Alveolar Artery; IOB, Infraorbital Artery; MA, Maxillary Artery; MAL, Malar Artery; OPR, Opthalmic Rete; PTG, Pterygoid Arterial Branches; RA, Ramus Anastomoticus; rDT, Rostral Deep Temporal Artery; SO, Supraorbital Artery.

The cranial arteries of the adult giraffe can be seen in Figs. 1–5 and Table S1. The cranial osteology of the giraffe is presented in Fig. S1.

### Branches of the external carotid artery

Branches of the external carotid artery are best seen in Figs. 1–3. The adult giraffe, as in other artiodactyls, lacks a patent proximal internal carotid artery (*arteria [a.] carotis interna*). This finding is somewhat consistent with that of Lawrence & Rewell (1948), who reported an internal carotid artery that does not interact with the cerebral arterial circle or carotid rete. It is more likely that their named internal carotid is the condylar artery, as it is reported to enter the cranium with the hypoglossal nerve, course caudally, and anastomose with the vertebral artery. Nomenclature aside, we find this morphology consistent. The absence of an internal carotid artery necessitates a somewhat arbitrary designation for the transition from the common carotid artery (*a. carotis communis*; CCA) to the external carotid artery (*a. carotis externa*; ECA). We define the ECA as a large distributing artery, originating immediately proximal to, and giving rise to, the occipital artery. The occipital artery (*a. occipitalis*) is therefore the first branch of the ECA (Figs. 1–3). Discrepancies in the literature variably refer to the artery traversing the lateral alar foramen (observed here in the juvenile giraffe) as the occipital artery. Lawrence & Rewell (1948) named the artery that passes through the alar foramen as the occipital because it is widely distributed throughout the cervical musculature in proximity to the atlas. Goetz & Keen (1957) report this vessel as the “descending occipital artery,” based on tentative homology with descending branch of the occipital artery of the ox. Rather than prescribing tentative homology, we elect to refer to the artery in question as the “alar artery” (*a. alaris*; new term), after its distribution. The occipital artery observed for the adult giraffe in this study is relatively small in size and branches from the dorsal surface of the ECA proximal to the occipital condyle (Figs. 1–3). Characteristically, the occipital artery ascends toward the nuchal region and occipital bone. Throughout its course, the occipital artery remains superficial to the bone, failing to leave a groove on the temporal crest as is present in camelids and smaller ruminants. In the adult giraffe, the occipital artery is not confluent with the condylar artery. Deep to the jugular process, a small ascending pharyngeal (*a. pharyngea ascendens*) vessel departs rostrally (Fig. 2). Immediately proximal to the condylar foramen, the ascending pharyngeal artery contributes the condylar artery (*a. condylaris*) and then continues to rostrally, following the basioccipital and dispersing throughout the pharyngeal muscles and mucosa (Figs. 2 and 3). The condylar artery traverses a short extracranial course to reach the condylar canal. Continuing dorsally, a large caudal meningeal artery (*a. meningea caudalis*) derives from the occipital artery, entering the skull through a larger, more medially placed mastoid foramen (within a shallow mastoid fossa; Fig. 2). The occipital artery reaches its apogee at the external occipital protuberance. Here, the contralateral vessels anastomose and form a single vessel that courses caudally, paralleling the nuchal ligament. Throughout its course, the occipital artery gives off comparatively few muscular branches (e.g., relative to other ruminants, suids, and camelids). The muscular branches that are present perfuse the musculature in close proximity to the occipital bone. Collateral muscle perfusion is accommodated by the robust alar artery. At no discernible point does the occipital artery, or its branches, directly or indirectly communicate with the cerebral arterial circle (*circulus arteriosus cerebri*; CAC; Fig. 3)—a substantial difference when compared to other ruminant artiodactyls. The condylar artery, frequently a derivative of the occipital artery, follows its namesake canal to reach the internal surface of the occipital condyle/foramen magnum (Fig. 3). Here a small caudal meningeal artery departs rostrally, while caudally, the condylar artery anastomoses with the vertebral artery (*a. vertebralis*). This closed loop prevents the vertebral arteries from conveying blood to the brain. This situation differs from that in many ruminant and suinamorph artiodactyls, through which the condylar artery communicates with the CAC via an anastomosis between the ventral spinal and basilar arteries (Daniel, Dawes & Prichard, 1953; Oliveira & Campos, 2005).

Next in succession, the lingual artery (*a. lingualis*) arises from the ventral surface of the ECA (Figs. 2 and 3). It is the largest rostroventral branch of the ECA and does not share a common trunk with the facial artery. Near its origin, the lingual artery contributes a variable number of laryngeal and pharyngeal arteries (*aa. laryngeae*; *aa. pharyngeae*) from its medial surface. Descending branches perfuse the larynx, along with ascending branches of the thyroid artery (*a. thyroidea*), and dorsal branches anastomose with derivatives of the lesser palatine artery (*a. palatina minor*) within the medial wall of the oropharynx. In the same segment, numerous parotid arterial branches (*aa. parotes*) arise from the lateral surface of the lingual artery before it continues rostrally along the ventral border of the hyoid apparatus. In close succession, the lingual artery contributes the hyoid arterial plexus (aa. hyoidei), supplying the muscles that suspend the hyoid. Rostroventrally, the lingual artery perfuses the mylohyoid muscle that encloses the floor of the oral cavity. Near the second mandibular molar, the lingual divides into the deep lingual and sublingual arteries (Figs. 1 and 3). The sublingual artery (*a. sublingualis*) is the ventral termination of the lingual artery (Fig. 3). This vessel courses rostrally and parallels the inner surface of the mandible until it terminates in the sublingual gland. Throughout its course, the sublingual contributes several lateral branches toward and around the ventral border of the mandible. The deep lingual artery (*a. profunda linguae*) is the dorsal termination of the lingual artery (Figs. 1 and 3). Within the parenchyma of the tongue, the deep lingual is highly dendritic to supply the intrinsic tongue musculature.

The course of the facial artery (*a. facialis*) is standard for artiodactyls (Fig. 1; Daniel, Dawes & Prichard, 1953; Sisson & Grossman, 1967). This vessel originates several centimeters distal to the larger lingual artery, and proximal to the caudal auricular artery. Near its origin, small branches ramify within the parotid gland. The facial artery courses ventrally until it reaches the angle of the mandible, after which it hooks around the bone to reach the superficial face. Immediately upon reaching the superficial tissues, the facial artery bifurcates into the mandibular and maxillary labial arteries (*a. labialis inferior* and *a. labialis superior*; Fig. 1). The mandibular labial artery is the rostral termination of the facial and parallels the mandible to distribute near the lower lip. The mandibular labial artery does not have a significant distribution rostral to the angle of the mouth, and the majority of the lower lip is supplied by the mental artery (a derivative of the maxillary artery via the inferior alveolar vessel). The maxillary labial artery is the dorsal continuation of the facial artery, and follows a tortuous path dorsally and rostrally, branching heavily throughout. There are few anastomoses with the dorsal nasal vessels on the lateral surface of the maxillary and nasal bones, and very few branches reach the upper lip, which is predominately supplied by the infraorbital artery (a derivative of the maxillary artery).

From the dorsal surface of the ECA, the caudal auricular artery (*a. auricularis caudalis*) arises immediately distal to the facial artery, slightly rostral to the jugular process (Figs. 1 and 2). This vessel courses obliquely across and superficial to the jugular process and mastoid region. As it passes over the mastoid, the stylomastoid artery (*a. stylomastoidea*) branches off from the caudal auricular artery. This vessel follows an extremely short extracranial course before entering the stylomastoid foramen, coursing through the facial canal, and supplying the middle ear and tympanum. At its dorsal termination, the caudal auricular artery divides into deep and medial auricular arteries (*a. auricularis profunda* and *a. auricularis medialis*) caudally. Rostrally, the vessel anastomoses freely with the superficial temporal and deep auricular vessels. The majority of the auricle is supplied through the caudal auricular artery, but collateral circulation is introduced via the rostral auricular artery—a derivative of the superficial temporal artery. This pattern is similar to that of other ruminants (Daniel, Dawes & Prichard, 1953; Sisson & Grossman, 1967).

The superficial temporal artery (*a. temporalis superficialis*; STA) is the last retromandibular branch of the superficial surface of the ECA (Figs. 1 and 2). The first two centimeters of this artery are essentially a common trunk shared between the transverse facial (rostral continuation), the STA (dorsal continuation), and the rostral auricular (caudal continuation) arteries. The STA crosses the zygomatic bone lateral to the TMJ. Immediately dorsal to the zygomatic, a small nutrient artery departs the parent vessel and courses rostrally over the superficial surface of the zygomatic to enter the bone at its contact with the temporal process of the postorbital bar. The STA then continues dorsally along the rostral margin of the temporalis muscle. A lateral palpebral artery arises at the level of contact between the temporal and zygomatic processes of the postorbital bar. This vessel divides into lateral superior and lateral inferior palpebral vessels at the outer margin of the orbit. Caudal to the superficial boundary of the orbit, the STA contributes the artery of the lacrimal gland (*a. lacrimalis*; Figs. 1 and 2). The dorsal continuation of the STA is the major supply of blood to the developing ossicone, and is homologous to the cornual artery (*a. cornualis*) of bovids, cervids, and *Antilocapra* (Figs. 1 and 2; Ganey, Ogden & Olsen, 1990; Davis, Brakora & Lee, 2011). The cornual artery enters the ossicone just below its midpoint, and prior to this gives off a large branch to supply the caudal aspect of the base of the horn. Collateral circulation to the ossicone is provided by the supraorbital artery (*a. supraorbitalis*; Figs. 1 and 2). Dorsally, an extensive superficial arterial plexus surrounds the ossicone, supplying the periosteum and the integument. These arteries contribute deeper branches to an intrinsic plexus (Figs. 1 and 3). The integument is therefore supplied by not only superficial arterial subsidiaries of the cornual and supraorbital vessels, but through anastomoses with the arteries supplying the ossicone internally. The intrinsic arterial plexus of this senescent individual extends throughout the entirety of the ossicone. This implies a difference between *G. camelopardalis* and the okapi, *Okapia johnstoni*—the only other living giraffid. In okapis, the apex of the ossicone has been observed as bare and polished, and the bone in this region may be avascular (Solounias, 2007; Hou et al., 2014), although our observations for the giraffe may be specific to mature females and not mature males. The ossicone itself is well-adhered to the frontal and parietal bones, and the periosteally-derived suture between the bony plates of the skull and the base of the ossicone is indistinct.

The transverse facial artery (*a. transversa facei*) is the first rostral branch of the STA (Figs. 1 and 2). It has a short distribution across the lateral face, with deeper branches piercing the superficial surface of the caudodorsal portion of the masseter muscle and superficial branches supplying the parotid gland. Unlike camelids and other ruminants, the giraffe transverse facial artery is situated well below the temporomandibular joint, and sends a long vessel dorsally to the joint capsule. Dorsal to the transverse facial artery, at the level of the condylar process, the deep masseteric artery (*a. masseterica profunda*) originates from the STA. The deep masseteric follows a short course deep to the masseter, in tight association with the mandibular bone. Its restricted distribution is to the caudodorsal quadrant of the masseter muscle.

The rostral auricular artery (*a. auricularis rostralis*) is the caudal termination of the STA (Fig. 2). Shortly before the rostral auricular artery crosses the zygomatic bone, a large vessel departs and courses dorsally to enter the temporal meatus through the large retroarticular foramen. This artery has been described as the middle meningeal artery (*a. meningea media*) in *Bos taurus* (Sisson & Grossman, 1967), but may be more appropriately named the temporal meningeal artery (new term). After exiting from the internal opening of the temporal meatus, dorsal to the petrosal, the middle (temporal) meningeal artery distributes across the caudolateral meninges. However, an artery originates from the CAC that distributes to the meninges underlying the temporal bone. This distribution pattern is in an analogous location to the middle meningeal artery of other mammals, but the origination from the CAC precludes inferences of homology. Before the rostral auricular artery reaches the retroarticular foramen, a small branch departs caudally and enters the middle ear. This is the rostral tympanic artery (*a. tympanica rostralis*). Finally, superficial branches of the rostral auricular supply the dorsal portion of the parotid gland and the ventral margin of the auricle. This vessel ramifies the caudal portion of the temporalis muscle before reaching the auricle. At the base of the auricle, the rostral auricular supplies a deep temporal vessel before its termination.

### Branches and distribution of the maxillary artery

The deeper branches and distribution of the maxillary are largely presented in Figs. 4 and 5, but many branches can also be seen in Figs. 1 and 3. Deep to the mandibular ramus, the inferior alveolar artery demarcates the transition from ECA to maxillary artery (*a. maxillaris*; MA). The inferior alveolar artery (*a. alveolaris inferior*) originates from the ventral surface of the MA between the superficial and deep temporal vessels (Figs. 3 and 5). The course is typical for mammals, entering the mandibular canal and supplying the roots of the mandibular dentition, and then terminating rostrally as the mental artery (*a. mentalis*; Figs. 1 and 3). The mental artery has an extensive distribution throughout the lower lip, partially ramified by the mandibular labial artery. Immediately dorsal to the inferior alveolar, several caudal deep temporal (*a. temporalis profunda caudalis*) branches arise (Figs. 4 and 5). The largest of these branches follows the surface of the temporal bone to supply the temporalis muscle from its deep boundary. This vessel is also the parent artery to the laterally-coursing masseteric artery (*a. masseterica*). The masseteric artery originates at the level of the mandibular incisure, through which it passes to reach its eponymous muscle. As it passes through the incisure, small branches ramify on the capsule of the temporomandibular joint. The MA then courses for several centimeters deep to the mandibular ramus without contributing major arterial branches (Figs. 3–5).

As the MA reaches the rostral border of the mandibular ramus, the arteria anastomotica originate from its dorsal surface. In a departure from standard artiodactyl morphology, the arteria anastomotica originate from a 1.5 cm common trunk, shared with the external ophthalmic artery (Figs. 4 and 5), instead of arising directly from the MA. These numerous anastomotic arterial branches form an extracranial, rostral continuation of the carotid rete (*rete mirabile epidurale rostrale*; CR; Figs. 4 and 5). Continuing this somewhat divergent pattern, the ramus anastomoticus derives from within the arteria anastomotica, anastomosing caudally with a direct branch from the MA (Figs. 3–5). This vessel courses caudally on the external surface of the basisphenoid, entering the cranium via the oval foramen. The internal course of the ramus anastomoticus is as expected: the vessel supplies the carotid rete and contributes a hypophyseal artery (*a. hypophysialis*). The carotid rete, and therefore the brain itself, receives the entirety of its oxygenated blood through the MA. For a thorough examination of the cerebral arterial circle and the distribution of blood to the giraffe brain, see Frąckowiak & Jakubowski (2008). The external ophthalmic artery (*a. ophthalmica externa*), although a derivative of the MA via the arteria anastomotica (Fig. 3), will be discussed in the following section with the blood supply to the eye and orbit.

On the ventrolateral surface of the MA, in close approximation to the external ophthalmic trunk, the buccal artery (*a. buccalis*) arises (Figs. 1 and 5). The buccal artery courses ventrolaterally through the pterygopalatine fossa, between the rostral border of the masseter muscle and the maxillary tuberosity. Before reaching the maxillary tuberosity, two arteries depart from the buccal artery: the rostral deep temporal artery (*a. temporalis profunda rostralis*) departs from the dorsal surface, and the pterygoid branches (*aa. pterygoidei*) depart the ventral surface (Figs. 4 and 5). The rostral deep temporal artery is a small vessel that arises proximal to the rostral border of the mandibular ramus and coronoid process. It follows a lengthy course on the deep surface of the temporalis muscle, beginning near the insertion of the muscle on the coronoid process and throughout the deep temporal fossa. The pterygoid branches extensively perfuse the medial and lateral pterygoideus muscles. One discrete branch from the plexus of pterygoid arteries enters the pterygoid bone as a nutrient artery. After the buccal artery exits the fossa between the masseter and maxillary tuberosity, the course of the vessel is typical for mammals: a dorsally coursing branch supplies the extraorbital fat pad and extends to the rostral border of the zygomatic arch, and a rostrally coursing branch supplies the buccinator muscle (Fig. 1). Throughout its facial course, the deep surface of the artery is extensively dendritic to supply the buccal glands. The buccal artery does not progress beyond the rostral margin of the orbit.

At its rostral termination, the MA splits into infraorbital, palatine, and malar arteries (Figs. 3 and 5). The infraorbital artery (*a. infraorbitalis*) is the most lateral of the two rostral continuations of the MA. From the pterygopalatine fossa, the infraorbital enters its eponymous canal (Figs. 1,  3,  4, 5). Within the maxillary bone, the artery distributes to the alveoli of the maxillary dentition. Upon exiting the infraorbital foramen, the artery has extensive divisions supplying the rostrolateral nasal region (Figs. 1 and 4). Its heavily dendritic termination is the predominant supply of the maxillary labium. Extracranially, the branches of the infraorbital anastomose copiously with terminal branches of the buccal and malar arteries (maxillary labial and caudal lateral nasal, respectively).

The malar artery (*a. malaris*) distributes to the lower eyelid and orbicularis oculi muscle, the ventral, medial, and dorsomedial margins of the orbit, the rostral frontal bone, and dorsal and caudal portions of the nasal region (Figs. 1 and 5). At the rostral boundary of the orbit, the malar departs from the infraorbital and courses dorsomedially. Near the ventral margin of the orbit, the medial inferior palpebral artery (*a. palpebralis inferior medialis*) departs laterally, along with several ventrally-coursing vessels bound for the orbicularis oculi muscle. As the malar continues dorsally, it departs into three heavily branching and anastomotic terminals. Caudally, the artery to the angle of the eye (*a. angularis oculi*) extensively perfuses the tissues overlying the dorsal margin of the orbit as well as the frontal bone. These branches provide collateral circulation to the superficial temporal and cornual (ossicone) arteries (Figs. 1 and 3). The rostral termination of the malar is the caudal lateral nasal artery (*a. lateralis nasi caudalis*), which anastomoses with terminal branches of the infraorbital artery rostrally, and the dorsal termination of the malar artery dorsally. The dorsal termination of the malar is the aptly named dorsal nasal artery (*a. dorsalis nasi*; Figs. 1 and 4).

The palatine vessels include the descending, greater, lesser, and sphenopalatine arteries from the termination of the MA, as well as an accessory lesser palatine artery that originates from the MA within the pterygopalatine fossa, and ramifies on the soft palate (Fig. 3). The descending palatine artery (*a. palatina descendens*) bifurcates from the MA, medial to the infraorbital artery (Figs. 3 and 5). This short artery contributes the remaining palatine vessels. The greater palatine artery enters the caudal palatine foramen and courses through the palatine canal, bifurcating proximal to the rostral palatine foramen (Fig. 3). The lower branch exits this foramen, coursing extracranially within the palatal grooves. Near the palatine fissure, the contralateral greater palatine arteries merge and continue rostrally to perfuse the rostral incisive bone and overlying keratinous pad. The internal branch supplies the floor of the nasal cavity. The lesser palatine artery emerges from the descending palatine and courses ventrally through the lesser palatine foramen and canal (Fig. 3). Within the mucosa covering the internal surface of the horizontal plate of the palatine, the lesser palatine artery (*a. palatina minor*) turns caudally and toward the midline (Fig. 3). Within the muscles and connective tissue of the soft palate, the right and left lesser palatine arteries unite and course caudally. The major branch of this vessel reaches the uvula, while lateral branches perfuse the soft palate and anastomose with pharyngeal vasculature (including direct derivatives of the lingual artery). The sphenopalatine artery (*a. sphenopalatina*) is the only dorsal branch of the descending palatine (Fig. 3). This vessel distributes extensively around the maxilloturbinates, with medial branches supplying the nasal septum.

### Arterial blood supply of the eye and orbital region

The majority of blood supplied to the orbital region is through the external ophthalmic artery (EO), a derivative of the MA (Figs. 4 and 5). This includes all intrinsic tissues, extraocular muscles, nerves, and the globe of the eye itself. Structures external or superficial to the orbit are collaterally supplied by the superficial temporal, buccal, and malar arteries. The external ophthalmic artery follows the same general pattern of other ruminants (Sisson & Grossman, 1967). The EO is a large branch from the dorsal surface of the MA (Figs. 4 and 5). It condenses from a common trunk with the rami anastomotica of the carotid rete. As such, its origin is at the caudal extent of the orbit near the foramen orbitorotundum. The EO courses to the apex of the orbit in close association with the dorsomedial orbitosphenoid. Throughout its course, it supplies the orbit, eye, periorbita, and extraocular muscles (with the exception of the lateral rectus muscle). There are two ophthalmic retia (*rete mirabile ophthalmica*) present: the superior rete is contiguous with the EO, whereas the inferior rete condenses from the rami anastomotica (Figs. 1, 3, 4 and 5). From these retia, branches condense to serve the globe of the eye. The ophthalmic rete receives the internal ophthalmic artery (from the CAC; *a. ophthalmica interna*). Distal to the ophthalmic rete, the EO re-condenses into several branches—several to the globe of the eye and sclera, as well as the external ethmoidal and supraorbital arteries. Smaller branches include the ciliary arteries (*aa. ciliares*) and the central artery of the retina (*a. centralis retinae*; which is ramified by a branch of the internal ophthalmic artery). The external ethmoidal artery (*a. ethmoidalis externa*) courses rostrally and dorsally along the median wall of the orbit, entering the cranium through the external ethmoidal foramen (Fig. 1). Internally, this artery supplies structures associated with the ethmoid bone, including the ethmoturbinals, ethmoidal air cells, and the caudal nasal septum. Across these structures, the external and internal ethmoidal arteries (*a. ethmoidalis interna*; derived from the cerebral circulation) anastomose freely. An anastomotic branch with the supraorbital artery is the dorsal continuation of the EO, and ascends to the internal supraorbital foramen in association with the orbitosphenoid and ethmoid bones. This artery has extensive branches inside the frontal air cells, supplying the epithelium of these moist, highly vascularized sinuses (Ganey, Ogden & Olsen, 1990; Mitchell & Skinner, 2003; Badlangana, Adams & Manger, 2011). After exiting the frontal sinus through the external supraorbital foramen, the artery continues as the frontal artery (*a. frontalis*), anastomosing with the dorsal nasal and superficial temporal/cornual arteries.

Extrinsic structures of the orbital region are not supplied by the EO, excepting structures of the frontal region supplied by the supraorbital artery. The lacrimal gland is supplied by the lacrimal artery (*a. lacrimalis*), a branch of the superficial temporal (Fig. 1). Caudal to the dorsolateral margin of the orbit, the lacrimal artery departs from the STA. The deep surface of the gland is also ramified by a small branch from the EO. The eyelids are supplied by lateral and medial sources. The lateral palpebral artery (*a. palpebralis lateralis*) is a long branch from the STA, beginning caudal to the postorbital margin at the level of contact between the frontal and zygomatic portions of the lateral orbit. Over this structure, the lateral palpebral artery divides into superior and inferior lateral palpebral arteries (*a. palpebralis superior lateralis* and *a. palpebralis inferior lateralis*). These supply the lateral two-thirds of the upper and lower eyelids and the lateral orbicularis oculi muscle. The medial half of the lower eyelid is supplied by the medial palpebral artery, which stems from the caudal surface of the malar near the ventral margin of the orbit. The infraorbital portion of the periorbital fat pad receives oxygenated blood from the buccal artery, and a portion of the supraorbital periorbital fat receives oxygenated blood from the deep temporal vessels (Fig. 1).

### Cranial arterial patterns of the juvenile giraffe

**Figure 6:**
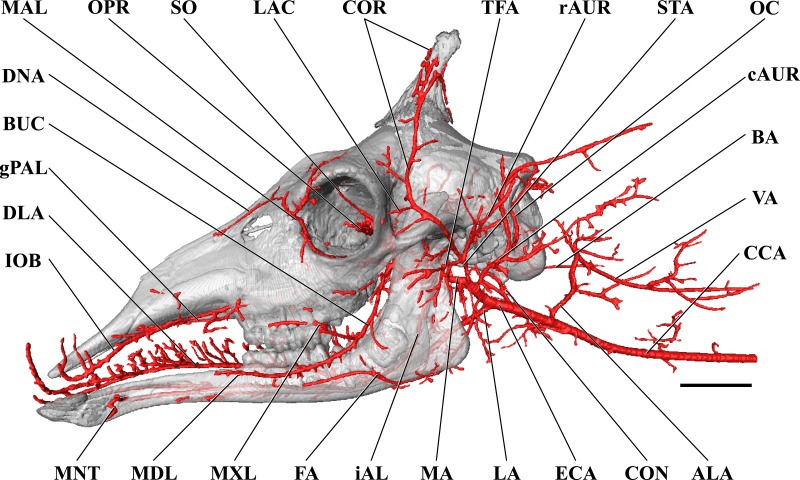
Cranial arteries of the juvenile (6 month-old) giraffe viewed from the lateral perspective. For clarity, smaller branches are unlabeled. These are referenced in Table S2. *Abbreviations*: ALA, Alar Artery; BA, Basilar Artery; BUC, Buccal Artery; cAUR, Caudal Auricular Artery; CCA, Common Carotid Artery; CON, Condylar Artery; COR, Cornual (ossicone) Artery; DLA, Deep Lingual Artery; DNA, Dorsal Nasal Artery; ECA, External Carotid Artery; FA, Facial Artery; gPAL, Greater Palatine Artery; iAL, Inferior Alveolar; IOB, Infraorbital Artery; LA, Lingual Artery; LAC, Lacrimal Artery; MA, Maxillary Artery; MAL, Malar Artery; MDL, Mandibular Labial Artery; MNT, Mental Artery; MXL, Maxillary Labial Artery; OC, Occipital Artery; OPR, Ophthalmic Retia; rAUR, Rostral Auricular Artery; SO, Supraorbital Arteries; STA, Superficial Temporal Artery; TFA, Transverse Facial Artery; VA, Vertebral Artery. Scale bar is 5 cm.

**Figure 7:**
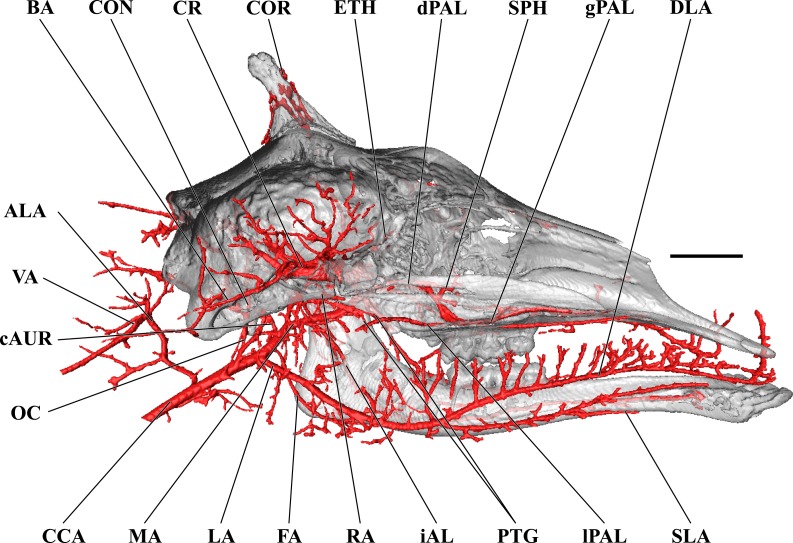
Cranial arteries of the juvenile giraffe, medial perspective. Cranial arteries of the juvenile (6 month-old) giraffe viewed from a midline sagittal section. For clarity, smaller branches are unlabeled. These are referenced in Table S2. *Abbreviations*: ALA, Alar Artery; BA, Basilar Artery; cAUR, Caudal Auricular Artery; CCA, Common Carotid Artery; CON, Condylar Artery; COR, Cornual Artery; DLA, Deep Lingual Artery; dPAL, Descending Palatine Artery; ETH, Ethmoidal Arteries; FA, Facial Artery; gPAL, Greater Palatine Artery; iAL, Inferior Alveolar; LA, Lingual Artery; lPAL, Lesser Palatine Artery; MA, Maxillary Artery; MNT, Mental; OC, Occipital Artery; PTG, Pterygoid Arterial Branches; RA, Ramus Anastomoticus; SLA, Sublingual Artery; SPH, Sphenopalatine Artery; VA, Vertebral Artery. Scale bar is 5 cm.

**Figure 8:**
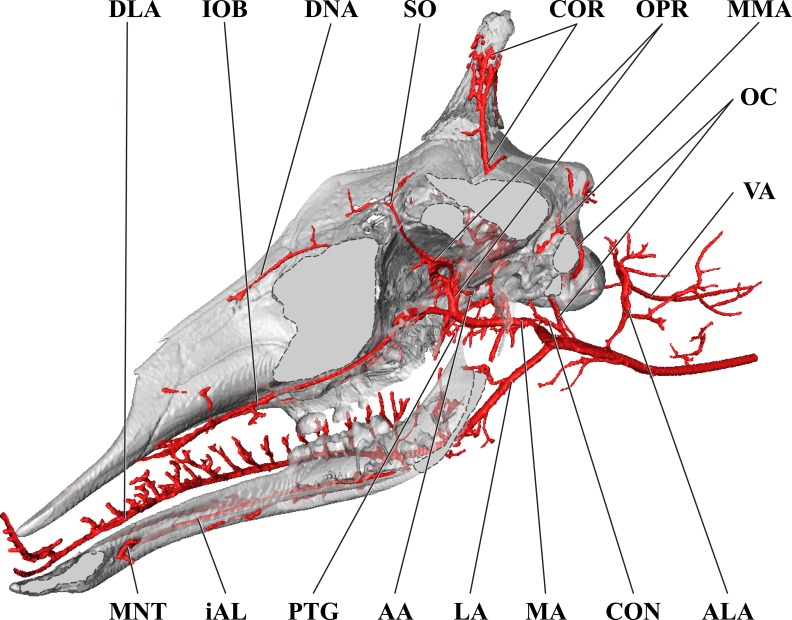
Orbital arteries and branches of the maxillary artery of the juvenile giraffe. Sagittal section of the juvenile (6 month-old) giraffe cranium, highlighting the orbital arteries and branches of the maxillary artery that are deep to the mandibular ramus. Grey blocks indicate plane of skull bone sections (clockwise from top): frontal and parietal bones; squamous portion of the temporal/zygomatic arch; mandible; maxillary bone. For clarity, smaller branches are unlabeled. These are referenced in Table S2. *Abbreviations*: AA, Arteria Anastomotica; ALA, Alar Artery; CON, Condylar Artery; COR, Cornual Artery; CR, Carotid Rete; DLA, Deep Lingual Artery; DNA, Dorsal Nasal Artery; IOB, Infraorbital Artery; LA, Lingual Artery; MA, Maxillary Artery; MNT, Mental Artery; MMA, Middle Meningeal Artery; OC, Occipital Artery; OPR, Ophthalmic Rete; PTG, Pterygoid Arterial Branches; SO, Supraorbital Artery; VA, Vertebral Artery. Scale bar is 5 cm.

**Figure 9:**
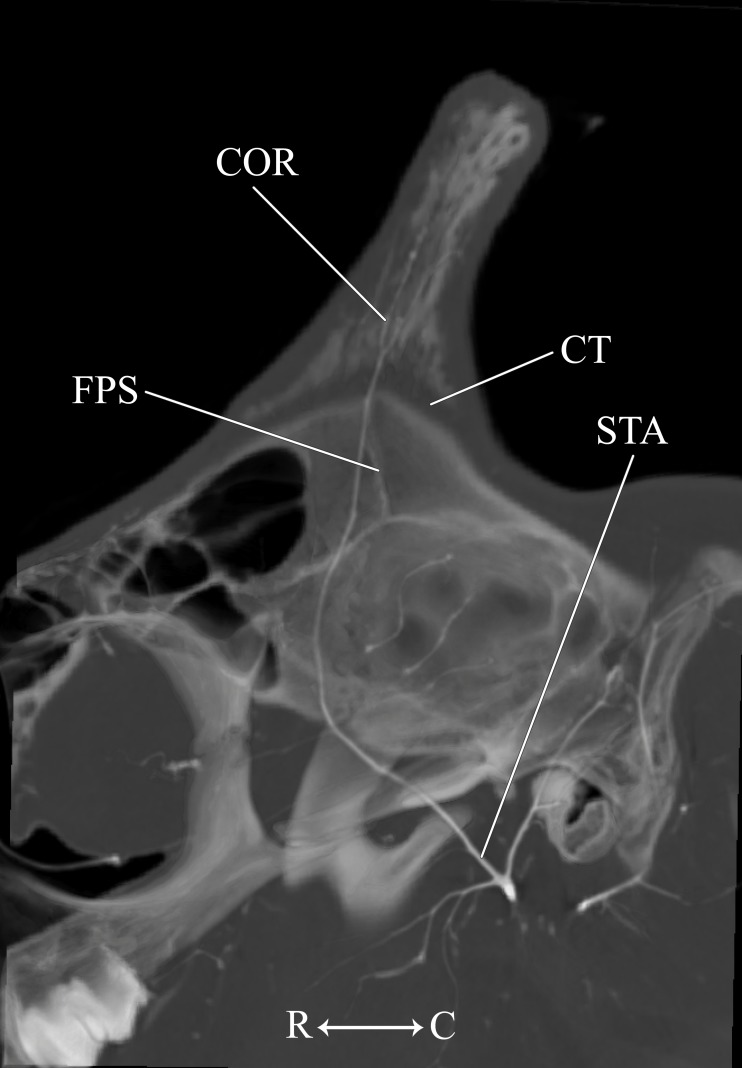
Developing ossicone of the juvenile giraffe. CT-scan section through the middle of the left ossicone. The ossicone of the juvenile giraffe is porous and highly trabeculated at the base. This texturing and the connective tissue lens underlying the developing ossicone, have been used to hypothesize that the ossicone develops from an epiphyseal plate. Here, a connective tissue (CT) lens is visible between the skull and the ossicone, however, the vascular invasion zone that is typical of physeal-type growth is conspicuously absent. *Abbreviations*: COR, Cornual Artery; CT, connective tissue lens; FPS, Frontoparietal Suture; STA, Superficial Temporal Artery. Note that a highly transparent volume rendering of the skull and arteries has been superimposed on the CT-scan section to enhance orientation.

The cranial arteries of the juvenile giraffe are presented in Figs. 6–8, and the developing ossicone in Fig. 9. See also Table S2 for a complete description of the juvenile giraffe arteries. The 6-month-old giraffe specimen included both the head and neck, providing an opportunity to describe the branches of the common carotid artery (CCA) lower in the neck (Figs. 6 and 8). Of particular interest is whether the CCA differs from the typical mammalian pattern, perhaps to accommodate the distinctively long neck of giraffes. The juvenile giraffe CCA follows the standard mammalian pattern until the artery reaches the atlas. At the midpoint of the atlas, a large artery departs from the dorsal surface of the CCA. This artery passes through the lateral alar foramen—a unique foramen that coincides with the alar fossa of other ruminants and Equidae (Figs. 4 and 6; Lawrence & Rewell, 1948). Due to this association, this artery is herein termed the “alar artery.” Within the alar foramen are two patent canals. The more lateral of these canals courses dorsoventrally and transmits a large derivative of the CCA to the neck muscles surrounding the atlas. Within the greater alar foramen, a large canal passes medially into the vertebral canal, through which a branch of the alar artery communicates directly with the vertebral artery. Although this anastomosis is present in other ungulates (Lawrence & Rewell, 1948; Sisson & Grossman, 1967), it is uniquely robust in the giraffe. Within the vertebral canal, the vertebral artery contributes a caudal meningeal artery, but does not otherwise connect to cerebral circulation. The vertebral artery terminates as an anastomosis with the condylar artery (Fig. 7). Because arteries do not have valves, blood can flow bidirectionally through such anastomoses. In this context, the alar-vertebral anastomosis of the giraffe has been implicated in mitigating significant blood pressure changes as the animal raises and lowers its head, especially considering the segregation from intracranial blood flow (Lawrence & Rewell, 1948; Mitchell & Skinner, 1993).

Other branches of the CCA include the cranial thyroid (*a. thyroidea cranialis*), descending pharyngeal (*a. pharyngea ascendens*), and external carotid arteries. The cranial thyroid artery branches from the ventral surface of the CCA near the departure of the aberrant alar artery. The vessel ascends in the neck to supply the thyroid gland. The descending pharyngeal artery branches immediately cephalad to the cranial thyroid artery. Within the walls of the esophagus and pharynx, the descending pharyngeal artery divides abundantly.

The CCA transitions to the ECA as defined for the adult giraffe and described above (demarcated by the occipital artery). The major caudal and deep cranial branches of the ECA are similar to the adult and will not be discussed in further detail (see Figs. 6–8). It is important to note that, in the 6-month-old giraffe, there is no evidence for a patent proximal segment of the internal carotid artery, suggesting the vessel obliterates earlier in development than reported for other large artiodactyls such as the domestic cow, *Bos taurus* (Gillilan, 1974; Zdun et al., 2014) and the water buffalo, *Bubalus bubalus* (Bamel, Dhingra & Sharma, 1975).

The arteries supplying the superficial face are highly disparate between the adult and juvenile specimens, particularly with regard to the facial and buccal arteries (Fig. 6). The facial artery departs the ventral surface of the ECA approximately 1 cm distal to the much larger lingual artery. In this specimen, the facial artery is reduced in size when compared with the adult giraffe. Near the origin of the facial artery, numerous small branches perfuse the parotid gland. From this point, the facial artery courses ventrally on the deep surface of the mandible, until it hooks under a notch rostral to the mandibular angle (Fig. 7). From here, the artery ascends for a short distance before following the mandible rostrally and terminating near the premolar row. The distribution of the juvenile giraffe facial artery is restricted to the proximal course typical of other ruminants (excepting caprine bovids), and does not contribute submental or rostral lateral nasal arteries (Fig. 6). These regions of the face are instead supplied by an enlarged buccal artery (derived from the MA). This condition may be unique to this specimen, as both this study and the only prior cranial arterial description of *Giraffa* to illustrate the superficial arteries of the face and head (Ganey, Ogden & Olsen, 1990) identify a well-developed facial artery. It should be noted that many artiodactyls either lack or have a truncated facial artery, with compensatory circulation deriving from branches of either the ECA or MA. The reduction of the facial artery is compensated for by the transverse facial artery, a derivative of the ECA, in the bovid subfamilies Caprinae (all members for which there are data; Sisson & Grossman, 1967; Daniel, Dawes & Prichard, 1953; Zdun et al., 2014 ), and Antilopinae (including *Antidorcas marsupialis* and *Saiga tatarica*; Zdun et al., 2014), as well as the family Camelidae (Nickel & Schwarz, 1963). As in this juvenile giraffe specimen, the buccal artery, a derivative of the MA, compensates for the absence of a facial artery in Suidae (*Sus scrofa*; Daniel, Dawes & Prichard, 1953; Nickel & Schwarz, 1963).

The atypical course of the buccal artery is similar to that described in suids (Fig. 6). The vessel courses through the pterygopalatine fossa, between the rostral border of the masseter and the caudal wall of the maxillary tuberosity. Before passing superficial to the maxillary tuberosity, two arteries depart from the buccal: the rostral deep temporal artery dorsally, and pterygoid branches ventrally. After exiting the fossa between the masseter and the maxillary tuberosity, a dorsally coursing branch of the buccal supplies the extraorbital fat pad and extends to the rostral border of the zygomatic where it branches heavily and terminates. The rostral continuation of the buccal supplies the buccinator muscle, but instead of terminating near the maxillary tuberosity, the distal course of this artery is considerably different from other artiodactyls. The distal buccal artery compensates for the reduced facial and transverse facial arteries branching from the ECA of this specimen. It terminates by bifurcating into mandibular and maxillary labial arteries, which perfuse the lips and surrounding tissues (Fig. 6). As in the adult, the maxillary labium receives the majority of its arterial blood from the infraorbital artery.

Other distinctions are related to the ontogeny of the cranial appendages. The base of the developing ossicone is highly porous and trabeculated (Fig. 9). This texture has been interpreted as a site of endochondral physeal growth (Spinage, 1968), and indeed we find that the developing ossicone sits above a lens of connective tissue, immediately dorsal to the frontoparietal suture. Although this connective tissue is radiolucent and cannot be identified to tissue type using CT scanning methodology, histological sectioning of similarly-aged juvenile giraffes performed by Ganey, Ogden & Olsen (1990) has identified this material as dense connective tissue with pockets of fibrocartilage. The function of these fibers is to adhere the ossicone to the underlying periosteum and bony plates of the skull, and not to serve as an endochondral anlage of the developing ossicone. Instead, ossification of the cranial appendage more likely develops through metaplastic ossification of dermally-derived fibrocellular connective tissue. Consistent with the observations of Ganey, Ogden & Olsen (1990), the surface of the skull below the horn is smooth. Moreover, contrary to the findings of Spinage (1968), we find that the porous base of the developing ossicone lacks an arterial plexus that would be consistent with the vascular invasion zone of endochondral ossification (Fig. 9). This finding supports the conclusions of Ganey, Ogden & Olsen (1990) that the connective tissue lens at the base of the developing ossicone is not homologous to the epiphyseal growth plates of developing long bones.

### Cranial arterial patterns of the neonate giraffe

**Figure 10:**
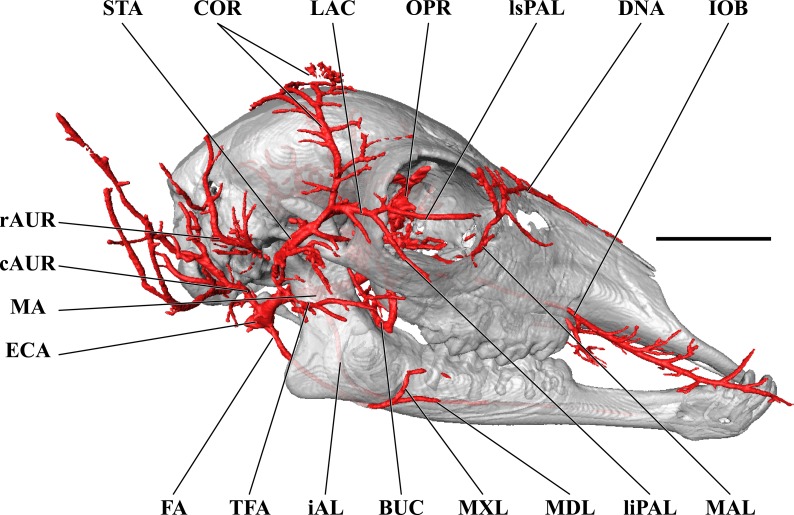
Cranial arteries of the neonate (stillborn) giraffe viewed from the lateral perspective. For clarity, smaller branches are unlabeled. These are referenced in Table S3. *Abbreviations*: BUC, Buccal Artery; cAUR, Caudal Auricular Artery; COR, Cornual (ossicone) Artery; DNA, Dorsal Nasal Artery; ECA, External Carotid Artery; FA, Facial Artery; gPAL, Greater Palatine Artery; iAL, Inferior Alveolar; IOB, Infraorbital Artery; LAC, Lacrimal Artery; liPAL, Lateral Inferior Palpebral Artery; lsPAL, Lateral Superior Palpebral Artery; MA, Maxillary Artery; MAL, Malar Artery; MDL, Mandibular Labial Artery; MNT, Mental Artery; MXL, Maxillary Labial Artery; OPR, Ophthalmic Retia; rAUR, Rostral Auricular Artery; SO, Supraorbital Arteries; STA, Superficial Temporal Artery; TFA, Transverse Facial Artery. Scale bar is 5 cm.

**Figure 11:**
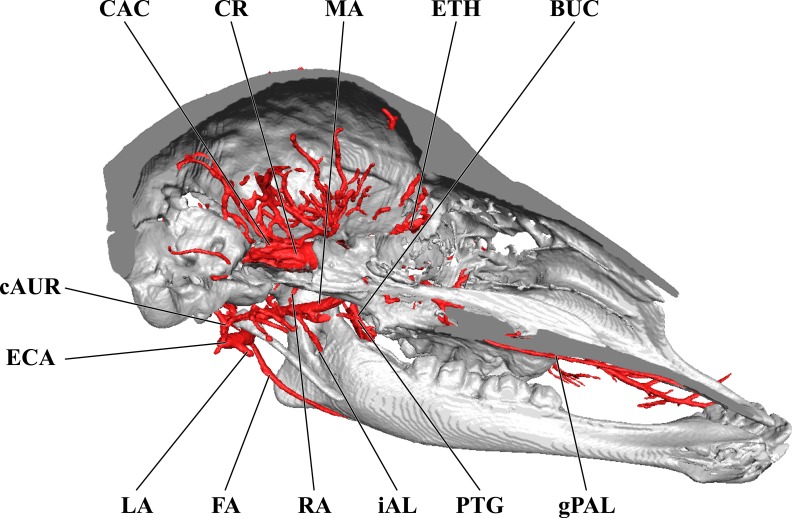
Cranial arteries of the neonate giraffe, medial perspective. Cranial arteries of the neonate (stillborn) giraffe viewed from a midline sagittal section. For clarity, smaller branches are unlabeled. These are referenced in Table S3. *Abbreviations*: BUC, Buccal Artery; CAC, Cerebral Arterial Circle (of Willis); cAUR, Caudal Auricular Artery; CR, Carotid Rete; ECA, External Carotid Artery; ETH, Ethmoidal Arteries; FA, Facial Artery; gPAL, Greater Palatine Artery; iAL, Inferior Alveolar; LA, Lingual Artery (cut); lPAL, Lesser Palatine Artery; MA, Maxillary Artery; PTG, Pterygoid Arterial Branches; RA, Ramus Anastomoticus. Scale bar is 5 cm.

**Figure 12:**
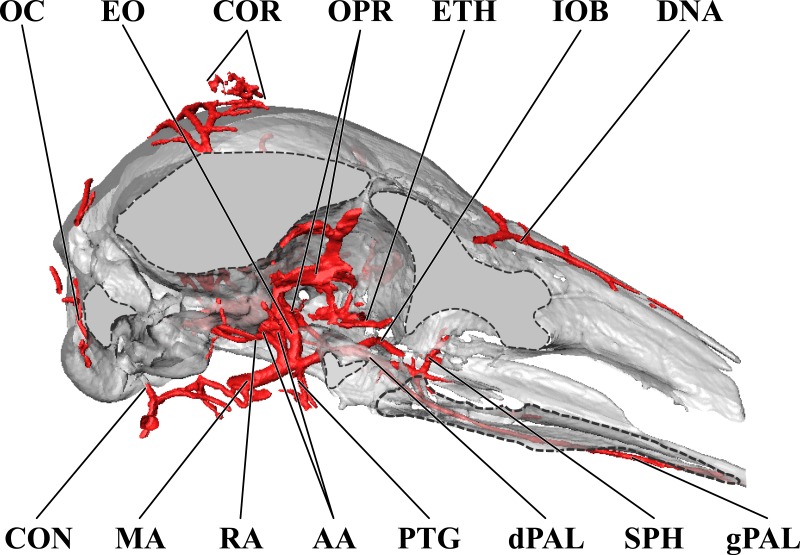
Orbital arteries and branches of the maxillary artery of the neonate giraffe. Sagittal section of the neonate (stillborn) giraffe cranium, highlighting the orbital arteries and branches of the maxillary artery that are deep to the mandibular ramus. Grey blocks indicate plane of skull bone sections (clockwise from top): frontal and parietal bones; squamous portion of the temporal/zygomatic arch; mandible; maxillary bone. For clarity, smaller branches are unlabeled. These are referenced in Table S2. Abbreviations: AA, Arteria Anastomotica; CON, Condylar Artery; COR, Cornual Artery; DNA, Dorsal Nasal Artery; dPAL, Descending Palatine Artery; EO, External Ophthalmic Artery; ETH, Ethmoidal Arteries; gPAL, Greater Palatine Artery; IOB, Infraorbital Artery; MA, Maxillary Artery; OC, Occipital Artery; OPR, Ophthalmic Rete; PTG, Pterygoid Arterial Branches; RA, Ramus Anastomoticus; SPH, Sphenopalatine Artery. Scale bar is 5 cm.

**Figure 13:**
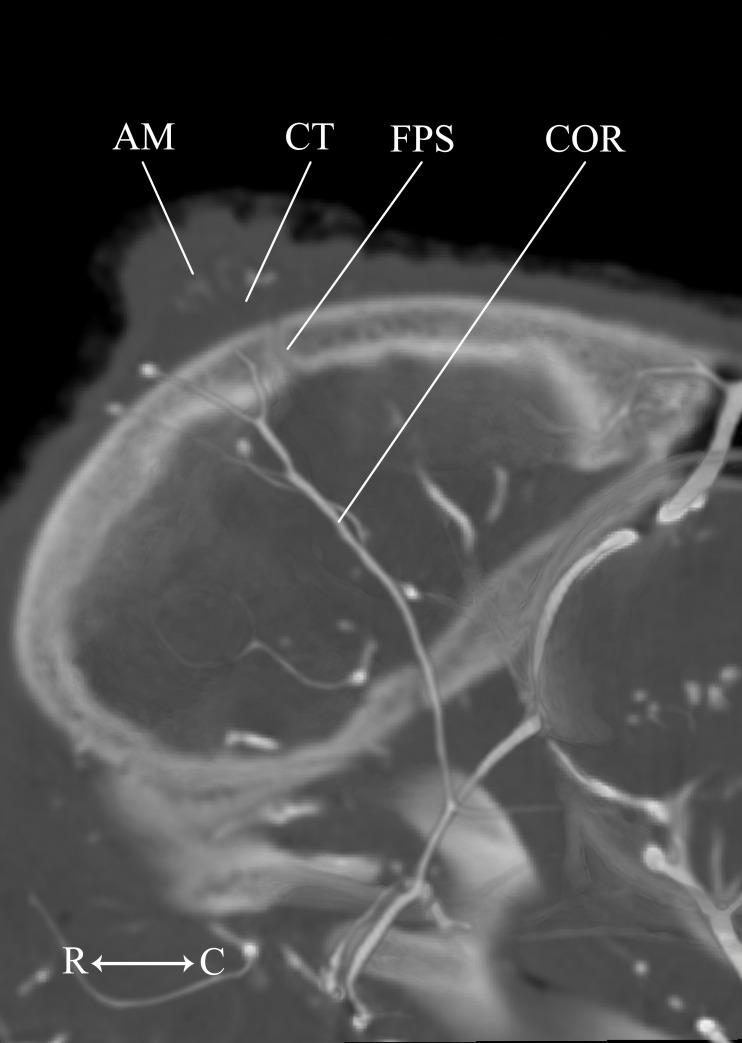
Developing ossicone of the neonate giraffe. CT-scan section through the middle of the right ossicone. The ossicone of the neonate has not yet begun to ossify and is comprised of connective tissue (CT) and a dense arterial mat (AM). This dense vascular mat within and underlying the developing ossicone may have contributed to early hypotheses that the ossicone develops from an epiphyseal plate. This arterial mat is not present in the developing ossicone of the juvenile. *Abbreviations*: AM, Arterial Mat; COR, Cornual Artery; CT, connective tissue lens; FPS, Frontoparietal Suture. Note that a highly transparent volume rendering of the skull and arteries has been superimposed on the CT-scan section to enhance orientation.

The cranial arteries of the neonatal giraffe are presented in Figs. 10–12, with the developing ossicone in Fig. 13. For complete details of the neonatal giraffe cranial arteries, see Table S3. The cranial arteries of the neonate giraffe are largely the same as the adult giraffe. In non-necropsied vessels, the arterial patterns of the neonate and adult giraffe overlap, and are consistent with the literature. Differences between the neonate specimen and the juvenile are mainly identified in the arterial supply to the facial region, and as discussed above, likely reflect pattern variation in the juvenile and are not a truly developmental sequence.

With respect to the juvenile, the arteries of the neck are presumed similar, but this specimen was sectioned very close to the base of the skull and the arteries of interest are not preserved. The main arterial tree begins at the occipital artery, close to the condylar foramen (Fig. 10). As in the juvenile and adult giraffe, there is no remnant of the ICA to demarcate division of the CCA into the ECA (Fig. 11). The majority of ECA branches are consistent between specimens in this growth series (Fig. 10). The occipital, caudal auricular, lingual, and superficial temporal arteries are consistent across all ontogenetic ages with respect to the location of departure from the ECA and the downstream branching patterns (Figs. 1, 6 and 10). The facial artery of the neonate is consistent with the distribution of this vessel in the adult giraffe (Figs. 1 and 10). Whereas the juvenile giraffe supplies much of the lateral facial region and the lips via the buccal artery (Fig. 6), the neonate follows the expected condition for ruminants (Fig. 10). From the rostroventral wall of the ECA, dorsal and deep to the angle of the mandible, the facial artery courses rostrally (Fig. 10). The vessel becomes superficial after passing a concavity created between the angle and body of the mandible. Near the developing 3rd lower molar, the facial artery bifurcates into rostral and superficial coursing branches. Similar to the adult giraffe (Fig. 1), the dorsal branch is substantial (Fig. 10). It is difficult to assess the distribution of the dorsal branch given the degree of necropsy, with the entirety of the buccal region transected. Based on the adult pattern, this vessel should reach and anastomose with the maxillary labial and dorsal nasal vessels. The rostral coursing termination should reach the mandibular labium to anastomose with the mental artery (derived from the inferior alveolar).

As expected, the extent of the buccal artery also differs between the neonate and juvenile specimens. The neonate buccal artery stems from the ventrolateral surface of the maxillary artery, in close proximity to the arteria anastomotica/external ophthalmic trunk. This artery courses laterally through the pterygopalatine fossa, between the rostral border of the masseter and the maxillary tuberosity (Fig. 10). Before reaching the maxillary tuberosity, the rostral deep temporal artery and pterygoid arteries depart, respectively, from the dorsal and ventral surfaces of the buccal artery. After exiting the fossa between the masseter and maxillary tuberosity, the proximal course of the buccal has a typical distribution, with dorsal branches supplying the extraorbital fat and the rostral border of the zygomatic arch, and a rostrally coursing branch supplying the buccinator muscle. The distribution of the buccal artery is to the deep muscles of mastication (medial and lateral pterygoideus muscles), the buccinator muscle, buccal fat, and the buccal glands. Smaller, caudal branches may supply the parotid gland, and the vessel does not continue rostrally beyond the rostral margin of the orbit.

The main arterial supply to the ossicone is derived from the same artery across developmental stages: the cornual artery, which is a derivative of the superficial temporal artery. The early stage ossicone does not receive significant collateral circulation, notably from the supraorbital arteries. The early development of the ossicone has been described as “physeal” type bone growth, but further examination has questioned this conclusion (Ganey, Ogden & Olsen, 1990). The developing ossicone can be seen as a radiolucent lens, situated above a dense arterial mat derived from the cornual artery (Fig. 13). The ventral surface of this arterial plexus is smooth, but the superficial surface evaginates the developing cartilage. Based on gross visualization alone, this vascular pattern superficially resembles the pattern expected of epiphyseal type bone growth. This visualization also helps to explain some of the early discrepancies regarding the mechanism of ossicone development, and highlights the importance of studying multiple developmental stages. Early studies of the perinatal ossicone identify this radiolucent connective tissue lens as the cartilaginous anlage for endochondral-type growth (Blumenbach, 1805; Murie, 1972; Spinage, 1968; Solounias, 1988). Without histological data, a vascular invasion zone would be the most parsimonious interpretation of the presence of this large arterial plexus deep to the connective tissue. Histological sections of the fetal ossicone (Ganey, Ogden & Olsen, 1990) indicate that this lens is fibrous connective tissue that gives rise to a well-vascularized, separate ossification center that develops above the skull before birth, but later fuses with the frontal and parietal bones throughout development. When the juvenile specimen is taken into consideration, it is clear that the arterial mat underlying the neonatal ossicone is not persistent even into early developmental stages.

The position of the cornual arterial mat ventral to the developing ossicone highlights a critical distinction between horn development in giraffes and bovid artiodactyls. The centers of ossification that contribute to horn growth in bovid artiodactyls (the os cornu) develop as intramembranous ossifications within the periosteum of the frontal bone (Dove, 1935; Ganey, Ogden & Olsen, 1990). In contrast, the ossicones of giraffes develop as centers of metaplastic ossification within the dermis (Ganey, Ogden & Olsen, 1990). The interposition of the cornual artery between the parietals and the ossicones is consistent with the expected course of the superficial temporal artery within the integument of the scalp, superficial to the periosteum of the parietals.

In all other respects, the arterial supply to the cranium of the neonate giraffe mirrors that of juvenile and adult developmental stages. The arteries of the superficial face and scalp, deep cranium, eye and orbital region, branches of the maxillary artery, and cerebral blood supply are consistent. Because the bone is not yet fully developed, there is very little interaction between the arteries and the ossifying skull. Foramina are beginning to form (e.g., the foramen orbitorotundum) and the concavity housing the carotid rete on the intracranial surface of the basisphenoid has formed, but no bony grooves are present.

**Table 1:** Major arterial discrepancies between successive giraffe developmental stages. Between all 3 stages represented, differences can be found in the following major distributing arteries: alar, facial, buccal, and cornual. The alar artery is presumed similar across all specimens, but is only preserved in the juvenile. The facial and buccal arteries of the neonate and adult are similar, whereas those of the juvenile follow patterns common to other artiodactyls. Finally, the cornual artery is the only major branch to show ontogenetic effects, first forming a plexus that underlies the developing ossicone in the neonate, then supplying the juvenile and adult ossicones through external and internal plexuses.

	Developmental stage		
Artery	Neonate	Juvenile	Adult
Alar	n/a	Originates from the dorsal surface of the common carotid and ascends to the lateral alar foramen of the atlas. Enters the foramen and ultimately anastomoses with the vertebral artery.	n/a
Facial	Departs the external carotid artery, courses dorsoventrally, and has significant distribution to the lower facial regions. Supplies the face superficial to the mandible and the maxillary and mandibular labia.	Departs from the ventral surface of the external carotid artery and courses rostroventrally. Reduced in size and does not continue rostrally beyond the mandibular premolar tooth row.	Departs the external carotid artery, courses dorsoventrally, and has significant distribution to the lower facial regions. Supplies the face superficial to the mandible and the maxillary and mandibular labia.
Buccal	Typical mammalian pattern to the buccinator muscle, buccal fat pad, and buccal glands.	Vessel is enlarged and compensates for reduced facial artery distribution. Gives rise to the mandibular and maxillary labial arteries.	Typical mammalian pattern to the buccinator muscle, buccal fat pad, and buccal glands.
Cornual	Forms a plexus directly underlying the developing ossicone.	Does not form a plexus directly underlying or significantly surrounding the developing ossicone.	Extends throughout the parenchyma surrounding the ossicone and forms a plexus within the bone itself.

## Conclusions

Cranial arterial development in the Masaai giraffe is conserved between stages. The majority of arteries supplying the brain and cranial base are consistent from the neonate to the senescent adult (summarized in Table 1). In our study, major contradictions between stages were only found within the facial vessels of the 6-month-old juvenile. For this specimen, the facial artery is more similar to that described for Caprinae, Antilopinae, and Camelidae (Nickel & Schwarz, 1963; Sisson & Grossman, 1967; Daniel, Dawes & Prichard, 1953; Zdun et al., 2014), and the buccal artery better follows descriptions for Suidae (Daniel, Dawes & Prichard, 1953; Nickel & Schwarz, 1963). The relative conservation of the giraffe cranial arteries between successive developmental stages differs from patterns of artiodactyl cranial arterial development available in the literature, particularly with regard to the internal carotid artery. In neonate suiformes (Wible, 1984), camelids (Lesbre, 1903; Tayeb, 1951; Kanan, 1970), and ruminants (Gillilan, 1974; Bamel, Dhingra & Sharma, 1975; Wible, 1984) a variable degree of patency is maintained in the external (proximal) segment of the internal carotid artery until it obliterates before maturity (Tandler, 1899; Tandler, 1906; Van Kampen, 1905; Daniel, Dawes & Prichard, 1953; Gillilan, 1974; Sisson & Grossman, 1967; Wible, 1984). Obliteration can occur early in development, as in pigs, sheep, goats, elk, and alpacas (Daniel, Dawes & Prichard, 1953; Gillilan, 1974; Sisson & Grossman, 1967; Wible, 1984), or close to sexual maturity, as in domestic cattle and buffalo (Gillilan, 1974; Bamel, Dhingra & Sharma, 1975). Early obliteration of the ICA is typical of smaller artiodactyls, but the neonate giraffe is born with a well-developed carotid rete and a completely closed ICA. Giraffes have the longest neck relative to body length among artiodactyls, and this may have developmental consequences. Among long-necked artiodactyls, giraffes have an approximately 20% longer gestation period (alpacas: 342 days (San-Martin et al., 1968); camels: 365–400 days (Eiwishy, 1987); giraffes: 400–460 days (Skinner & Hall-Martin, 1975)), so the cranial arteries may be more fully developed by parturition. The ontogenies of major artiodactyl groups show considerable variation in the timing and extent of ICA obliteration and carotid rete proliferation. These differences could have important implications for the development and evolution of this vital thermoregulatory structure and the remarkable success of Artiodactyla.

## Supplemental Information

10.7717/peerj.1696/supp-1 Table S1 Cranial arteries of the adult giraffe

10.7717/peerj.1696/supp-2 Table S2 Cranial arteries of the juvenile giraffe

10.7717/peerj.1696/supp-3 Table S3 Cranial arteries of the stillborn giraffe

10.7717/peerj.1696/supp-4 Figure S1 Abbreviated osteology of the giraffe skull

## Additional Information and Declarations

### Data Availability

The following information was supplied regarding data availability: Datasets are 3D surface renderings saved as .stl format. .stl’s can be read using a number of free surface viewers, including MeshLab (freeware): http://meshlab.sourceforge.net/
Adult Giraffe Skull: 10.6084/m9.figshare.2009739
Adult Giraffe Arteries: 10.6084/m9.figshare.2009742
Juvenile Giraffe Skull: 10.6084/m9.figshare.2009745
Juvenile Giraffe Arteries: 10.6084/m9.figshare.2009748
Neonate Giraffe Skull: 10.6084/m9.figshare.2009751
Neonate Giraffe Arteries: 10.6084/m9.figshare.2009754.
