# Supplementary material for: A comparison of postnatal arterial patterns in a growth series of giraffe (Artiodactyla: Giraffa camelopardalis)
Source: PeerJ. 2016 Feb 16;4:e1696. doi: 10.7717/peerj.1696 (PMC4768699; doi:10.7717/peerj.1696)
Supplement: Table S1 [file peerj-04-1696-s001.pdf]

Cranial Arteries of the Adult Giraffe

Branches of the External Carotid Artery

Artery	Origin	Course	Distribution
External Carotid	Common Carotid @ occipital	Arbitrarily begins at occipital artery; as in most artiodactyls, the giraffe does not have an internal carotid artery to demarcate the termination of the common carotid artery.	Carries entirety of oxygenated blood to the head.
Occipital	External Carotid	First branch of the external carotid. Branches from the dorsal surface distal to the alar artery. Ascends toward the occipital bone, following a pathway that is largely obscured by the jugular process. A caudal meningeal branch enters the mastoid foramen, within mastoid fossa. At the external occipital protuberance, unifies with contralateral occipital artery. This single vessel courses caudally, paralleling the nuchal ligament.	Throughout its course, the occipital artery gives off few muscular branches. These perfuse the musculature in close proximity to the occipital bone. Collateral muscular perfusion is accommodated by the alar artery. Additional distribution is to the caudal meninges.
Condylar	External Carotid	Short extracranial course to reach condylar canal. On internal surface of occipital condyle, a small caudal meningeal artery departs. The condylar then immediately courses caudally to anastomose with the vertebral artery. Does not contact cerebral arterial circle.	Distributes only to caudal meninges and vertebral artery.
Caudal Auricular	External Carotid	Originates anterior to the jugular process, following the condylar artery in succession. Courses obliquely across and superficial to jugular and mastoid, does not contact the mastoid, although dorsal continuation grooves the nuchal crest. Bifurcates into caudal and medial auricular arteries posteriorly; anastomoses with superficial temporal and deep auricular vessels anteriorly.	Majority of auricle supplied through caudal auricular.
Stylomastoid	Caudal auricular	Extremely short extracranial course before entering stylomastoid foramen. Courses through facial canal to supply middle ear and tympanum.	Middle ear, tympanum.
Deep Auricular	Caudal Auricular	Dorsal termination of caudal auricular; after supplying the base of the auricle, penetrates caudal border of the temporalis muscle.	Base of auricle, temporalis muscle.

Lingual	External Carotid	Largest rostroventral branch of the external carotid. Proximate to its origin, contributes laryngeal and pharyngeal branches medially and dorsally, and parotid (glandular) branches laterally. Superior branches anastomose with lesser palatine. Continues rostrally along ventral border of hyoid apparatus. Contributes hyoid plexus, supplying the muscles that suspend the hyoid. Rostrally, perfuses the mylohyoid muscle to enclose floor of mouth. Near the second lower molar, divides into deep and sublingual branches.	Parotid, pharynx, larynx, hyoid, floor of mouth, mylohyoid, parenchyma of tongue, submandibular gland and sublingual gland.
Deep Lingual	Lingual	Departs dorsal surface of lingual artery near the second lower molar. Highly dendritic within the parenchyma of the tongue.	Tongue and intrinsic muscles.
Submental	Lingual	Lingual artery bifurcates near the ventral border of the mandible, proximate to the molar tooth row.	Submental gland; oral mucosa.
Sublingual	Lingual	Ventral terminal branch of lingual. Courses rostrally and parallels inner surface of the mandible to reach the sublingual gland. Rostral termination is at sublingual gland.	Sublingual gland.
Facial	External Carotid	Originates following the larger lingual artery. Near its origin, small branches contribute to parotid. Courses ventrally until angle of mandible; hooks under notch rostral to angle to course dorsally and immediately bifurcates into mandibular and maxillary labial vessels. Mandibular labial parallels mandible to distribute near lower lip; maxillary labial follows tortuous path dorsally and rostrally. Mandibular labial branches heavily throughout.	Parotid gland; Face below maxillary tuberosity; lower and upper lips (although these are predominately supplied by other arteries).
Mandibular Labial	Facial	Rostral termination of the facial artery.	Caudal portion of lower lip, near angle of the mouth. Rostrally, the lower lip is supplied by the mental artery.
Maxillary Labial	Facial	Dorsal continuation of facial; extensively dendritic within face; however, few anastomoses on lateral nasal surface. Very few tributaries reach the upper lip, which is predominately supplied by the infraorbital artery.	Lateral facial region, superficial to the maxillary dentition; large vessel with extensive distribution.

Superficial Temporal	External Carotid	Last retromandibular branch of the external carotid. After short course, transverse facial departs rostrally. Following departure of the transverse facial, the superficial temporal continues dorsally. The rostral auricular departs in the caudal direction, near the mandibular condyle and branches heavily throughout rostral auricle. The superficial temporal crosses zygomatic arch lateral to the temporomandibular joint. At level of contact between the temporal and zygomatic processes of the frontal bone arises a lacrimal/lateral palpebral artery. Divides into lateral superior and lateral inferior palpebral vessels over post-orbital bar. Caudal to the dorsal boundary of the orbit, supplies lacrimal gland via eponymous branch.	Supplies the face near the masseter muscle, auricle, lacrimal gland, eyelid, temporalis muscle, and ossicone.
Transverse Facial	Superficial Temporal	First rostral branch of the superficial temporal, originating proximal to the rostral auricular. Short distribution across lateral face. Deeper branches pierce the superficial surface of caudodorsal masseter m., superficial branches supply the parotid gland.	Masseter muscle, parotid gland.
Lacrimal	Superficial Temporal	Caudal to the superolateral margin of the orbit, the lacrimal artery departs from the superficial temporal. Supplies the lacrimal gland; terminates as lateral palpebral vessels.	Lacrimal gland; palpebrae.
Lateral Superior Palpebral	Superficial Temporal	Originates as termination of lacrimal artery; shares a common trunk with the lateral inferior palpebral at the level of contact between the frontal and zygomatic portions of the lateral orbital bar. Supplies upper palpebrum lacrimal gland, and lateral orbicularis occuli muscle.	Superior palpebrum, lacrimal gland, and orbicularis oculi muscle.
Lateral Inferior palpebral	Superficial Temporal	Ventral termination of the lacrimal artery, coursing toward and within the inferior palpebrum.	Inferior palpebrum and orbicularis oculi muscle.
Anterior Tympanic	Superficial Temporal	Enters middle ear after branching from the posterior surface of the superficial temporal, before the parent artery nears the post glenoid foramen.	Tympanum.
Middle Meningeal	Superficial Temporal	Enters temporal meatus via post glenoid foramen. Distributes across posterolateral meninges after exiting the internal opening of the temporal meatus, superior to the petrosal.	Posterolateral meninges.

Cornual (Ossicone)	Superficial Temporal	Dorsal termination of the superficial temporal, and the major artery supplying ossicone. Extensive superficial plexus surrounds ossicone and contributes deeper branchs to internal plexus. Rostral branches ramify the dorsal nasal artery on the lateral surface of the median frontal promenance. Collateral circulation to ossicone is via extensive anastomosis with the supraorbital vessels.	Ossicone and frontal/parietal bones surrounding the base of the ossicone.
Rostral Auricular	Superficial Temporal	Caudal termination of superficial temporal, arising distal to the transverse facial artery. Courses caudally, with superficial branches supplying the dorsal portion of the parotid gland and the ventral margin of the auricle. Ramifies caudal portion of temporalis muscle before reaching the auricle. At base of auricle, supplies a deep temporal vessel and auricular base. Terminates near base of auricle.	Parotid gland, temporalis muscle, auricular base, auricle.
**Branches of the Maxillary Artery**			
Inferior Alveolar	Maxillary	Originates from ventral surface of the maxillary artery, between the superficial and deep temporal vessels. Courses laterally to enter the mandibular canal, wherein branches depart for the alveoli of the mandibular dentition. Terminates superficially as the mental artery, and anteriorly by supplying the lower incisors.	Roots of mandibular dentition; continues as mental artery to supply inferior labium.
Mental	Inferior Alveolar	Rostral continuation of the inferior alveolar artery. Exits mandibular canal through the mental foramen, before continuing rostrally to serve as the predominant source of oxygenated blood to lower lip.	Inferior labium; partially ramified by sublingual artery.
Caudal Deep Temporal	Maxillary	Direct tributary of the maxillary artery that arises immediately dorsal to the inferior alveolar. Follows the surface of the temporal bone to supply the temporalis muscle from its deep border. Parent artery contributes laterally-coursing masseteric artery at the level of the mandibular incisure.	Temporalis and masseter muscles.
Masseteric	Caudal Deep Temporal	Traverses mandibular incisure (between coronoid and condylar processes) to immediately supply masseter. As it passes through the mandibular incisure, a small branch ramifies the temporomandibular joint.	Masseter muscle, temporomandibular joint.
Ramus Anastomoticus	Maxillary	Arises from the maxillary artery, ascends toward basicranium and enters via the foramen ovale to ramify the carotid rete.	Carotid rete.

Buccal	Maxillary	Stems from ventral surface of the maxillary artery, in close proximity to the arteria anastomotica. Courses through the pterygopalatine fossa, between the rostral border of the masseter and the maxillary tuberosity. Before reaching the maxillary tuberosity, two arteries depart from the buccal: the rostral deep temporal artery dorsally, and pterygoid branches ventrally. The origin of the pterygoid branches is nearly shared with the MA. Once the buccal has reached the superficial face, a dorsally coursing branch supplies the extraorbital fat and extends to the rostral border of the zygomatic, and a rostrally coursing branch supplies the buccinator muscle. The buccal artery does not progress beyond the rostral margin of the orbit.	Periorbita, buccinator muscle.
Pterygoid Branches	Maxillary/Buccal	Near contact between the buccal and the maxillary arteries, pterygoid branches depart from ventral surface of the buccal artery. Supplies pterygoid muscles from caudal border. Extensively dendritic within muscular parenchyma. Rostral vessel enters pterygoid bone as a nutrient artery.	Pterygoid muscles and bone.
Rostral Deep Temporal	Buccal	Small artery originating from the superficial surface of the buccal artery, proximal to the rostral border of the coronoid process. Lengthy course on deep surface of the temporalis muscle, beginning near the muscle's insertion on the coronoid and throughout the deep temporal fossa.	Temporalis muscle and tendon.
Arteria Anastomotica	Maxillary	Originates near the external ophthalmic artery, within the external rostral continuation of the carotid rete. Courses caudally and enters the cranium via the foramen ovale. Supplies caudal carotid rete. Contributes hypophyseal vessel.	Carotid rete.
External Ophthalmic	Maxillary/rami anastomotica	The external ophthalmic artery is a large tributary from the dorsal surface of the maxilliary artery. It shares a common trunk with the rami anastomotica, as such its origin is close to the foramen orbitorotundum. The EO courses to the apex of the orbit in close association to the orbitosphenoid. Its tributaries are described below (see: *Arterial Supply to the Eye and Orbit*).	Majority of orbit, eyeball, and periorbita.
Infraorbital	Maxillary	Rostral continuation of the maxillary. From the pterygopalatine fossa, enters the infraorbital canal and distributes to the alveoli of maxillary dentition. Becomes superficial via the infraorbital foramen and contributes to rostrolateral nasal and maxillary labial circulation.	Alveoli of cheek teeth, nasal and maxillary labial soft tissues.

Malar	Infraorbital	At the rostral border of the orbit, the malar departs the infraorbital and courses dorsomedially. Near the ventral margin of the orbit, the medial inferior palpebral departs laterally, along with several ventrally-coursing vessels bound for the orbicularis oculi muscle. As the malar continues dorsally, it departs into 3 heavily branching and anastomotic terminals. Caudally, the artery to the angle of the eye extensively perfuses the tissues overlying the dorsal margin of the orbit and the frontal. It anastomoses with the superficial temporal/cornual. The rostral termination is the caudal lateral nasal, which anastomoses with the rostral lateral nasal rostrally and the dorsal termination of the parent artery dorsally. The dorsal termination is the aptly named dorsal nasal artery.	Lower eyelid and orbicularis oculi m., ventral, medial, and dorsomedial margins of the orbit; rostral frontal bone; dorsal and caudal portions of the nasal region.
Rostral Lateral Nasal	Malar	Dorsal termination of the malar; Courses caudally toward ossicone. Provides collateral circulation to base of ossicone and anastomoses freely with deeper branches of the superficial temporal.	Apex of nasal region; dorsal margin of orbit; base of ossicone; deep rostral border of temporalis muscle.
Dorsal Nasal	Malar	Malar ascends near ventromedial wall of orbit, turns medial at lacrimal fossa and continues as lateral nasal. Near its origin, several branches pierce the skull to enter the extensive frontal sinuses.	Lateral nasal region; mucosa of frontal sinuses.
Descending Palatine	Maxillary	Rostral termination of the maxillary artery- arises as a bifurcation with the infraorbital. Contributes greater, lesser, and sphenopalatine arteries.	Hard and soft palates, nasal mucosa and maxilloturbinates.
Accessory Lesser Palatine	Maxillary	Small branch distal to terminal bifurcation of maxillary artery; ramifies soft palate.	Rostral soft palate.
Lesser palatine	Descending palatine	From descending palatine, courses ventrally through lesser palatine foramen/canal. Within muscles and connective tissue of soft palate, courses caudally and toward midline on internal surface of horizontal palatine. At midline, joins contralateral lesser palatine. Continues caudally with major branch reaching the uvula and lateral branches that ramify the pharyngeal vasculature (including direct derivatives of lingual artery).	Soft palate, pharynx.

Greater Palatine	Descending palatine	Enters caudal palatine foramen and courses through palatine canal. Bifurcates before lower branch exits via rostral palatine foramen. Rostral branch remains internal and supplies floor of nasal cavity. External branch courses in palatal grooves. Near the palatine fissure, joins the contralateral greater palatine and continues rostrally to anterior incisive and keratinous pad.	Nasal floor, hard palate and associated structures, incisive and keratinous pad.
Sphenopalatine	Descending palatine	Only dorsal branch of descending palatine. Distributes extensively around maxilloturbinates; medial branches supply nasal septum.	Maxilloturbinates; nasal septum.
**Arterial Supply to the Eye and Orbit**			
External Ophthalmic	Maxillary / Rami Anastomotica	The external ophthalmic artery is a large branch from the dorsal surface of the maxilliary artery. It shares a common trunk with the arteria anastomotica; as such its origin is close to the foramen orbitorotundum. The external ophthalmic courses to the apex of the orbit in close association to the orbitosphenoid. Throughout its course, it supplies the majority of structures associated with the eye and orbit, including the extraocular muscles (not lateral rectus). Receives anastomosis from internal ophthalmic. Ophthalmic retia are restricted to the external ophthalmic artery, and are not confluent with the carotid rete or arteria anastomotica. Branches condense from the ophthalmic rete to serve the globe of the eye. Terminates by bifurcating into external ethmoidal and supraorbital arteries.	Majority of orbit, eyeball, and periorbita.
Internal Ophthalmic	Cerebral Arterial Circle	Courses through the optic foramen and joins the ophthalmic rete. Note: the internal ophthalmic does not directly contact eye or orbital structures.	Connects the cerebral arterial circle to ophthalmic rete; does not directly supply eye.
Central Artery of the Retina	Infraorbital artery	Small artery. From the infraorbital, the central artery of the retina pierces the dura and connective tissue sheath of the optic nerve near the origin of the nerve from the optic chiasm. Courses within nerve tissue to supply retina.	Retina.
Lacrimal	Superficial Temporal	Caudal to the superolateral margin of the orbit, the lacrimal artery departs from the superficial temporal. Supplies the lacrimal gland; terminates as lateral palpebral vessels.	Lacrimal gland.

Lateral Superior Palpebral	Superficial Temporal	Originates as termination of lacrimal artery; shares a common trunk with the lateral inferior palpebral at the level of contact between the frontal and zygomatic portions of the lateral orbital bar. Supplies upper palpebrum lacrimal gland, and lateral orbicularis occuli muscle.	Superior palpebrum, lacrimal gland, and orbicularis oculi muscle.
Lateral Inferior palpebral	Superficial Temporal	Ventral termination of the lacrimal artery, coursing toward and within the inferior palpebrum.	Inferior palpebrum and orbicularis oculi muscle.
