# Supplementary material for: A comparison of postnatal arterial patterns in a growth series of giraffe (Artiodactyla: Giraffa camelopardalis)
Source: PeerJ. 2016 Feb 16;4:e1696. doi: 10.7717/peerj.1696 (PMC4768699; doi:10.7717/peerj.1696)
Supplement: Table S2 [file peerj-04-1696-s002.pdf]

Cranial Arteries of the Juvenile Giraffe

Branches of the External Carotid Artery

Artery	Origin	Course	Distribution
External Carotid	Common Carotid Artery @ Occipital	Arbitrarily begins at occipital artery; as in most artiodactyls, the juvenile giraffe does not have an internal carotid artery to demarcate the termination of common carotid.	Carries entirety of oxygenated blood to the head.
Occipital	External Carotid	First branch of the external carotid; branches from dorsal surface near tip of jugular process. Ascends toward condylar foramen/occipital bone. Deep to jugular process, an artery departs rostrally toward the pterygoid canal. Before reaching the condylar foramen, the occipital contributes condylar artery. The occipital then follows the caudal surface of nuchal/temporal crest, superficial to the bone. *Small* deep stylomastoid vessel enters braincase via small, lateral mastoid foramen. Caudal meningeal branch enters larger mastoid foramen within mastoid fossa. At the external occipital protuberance, the contralateral occipital arteries unite, and this single vessel courses caudally, paralleling the nuchal ligament.	Throughout its course, the occipital artery gives off few muscular branches. These perfuse the musculature in close proximity to the occipital bone. Collateral muscular perfusion is accommodated by the alar artery. Additional distribution is to the caudal meninges and vertebral artery (via the condylar).
Condylar	Occipital	Short extracranial course to reach condylar canal. On internal surface of occipital condyle, a caudal meningeal artery departs before the condylar courses caudally to anastomose immediately with vertebral artery. Does not contact cerebral arterial circle.	Distributes only to caudal meninges and vertebral artery.
Caudal Auricular	External Carotid	3cm distal to occipital artery; begins anterior to jugular process, courses obliquely across and superficial to jugular process and mastoid, except for a groove left where the squamous temporal contacts the mastoid (posterior to the external acoustic meatus). Bifurcates into caudal and medial auricular arteries posteriorly; anastomoses with superficial temporal and deep auricular vessels anteriorly.	Majority of auricle supplied through caudal auricular.
Stylomastoid	Caudal auricular	Extremely short extracranial course before entering stylomastoid foramen. Courses through facial canal to supply middle ear and tympanum.	Middle ear, tympanum.
Deep Auricular	Caudal Auricular	Dorsal termination of caudal auricular; after several centimeters, penetrates caudal temporalis muscle.	Base of auricle, temporalis muscle.

Lingual	External Carotid	Largest rostroventral branch of external carotid. Proximal to its origin, contributes laryngeal and pharyngeal branches medially and dorsally, and parotid branches laterally. Dorsal branches anastomose with lesser palatine near isthmus fauces. Continues rostrally along ventral border of hyoid apparatus where it contributes the hyoid plexus, supplying the muscles that suspend the hyoid. Rostrally, perfuses the mylohyoid muscle to enclose floor of mouth. Near the second lower molar, divides into deep and sublingual branches.	Parotid gland, pharynx, larynx, hyoid, floor of oral vestibule, mylohyoid, parenchyma of tongue, submandibular gland, and sublingual gland.
Deep Lingual	Lingual	Dorsal termination of the lingual artery. Highly dendritic within the parenchyma of the tongue.	Tongue and intrinsic muscles.
Sublingual	Lingual	Ventral terminal branch of lingual. Courses rostrally and parallels inner surface of the mandible to reach the sublingual gland. Throughout its course, contributes several lateral branches toward and around the mandible. Rostral termination is at sublingual gland.	Sublingual gland.
Facial	External Carotid	Reduced in size relative to other artiodactyls. Originates distal to larger lingual artery; proximal to caudal auricular. Near its origin, small branches contribute to parotid. Courses ventrally until angle of mandible; hooks under notch rostral to angle, ascends for short distance before following the mandible rostrally.	Distribution restricted to proximal course of typical giraffe facial artery. Compensatory flow is accomplished by the buccal artery. Supplies parotid gland and skin/superficial muscles of proximal lower jaw. Does not contribute labial, submental or rostral lateral nasal vessels.
Mandibular Labial	Buccal	Inferior termination of the enlarged buccal artery.	Caudal portion of inferior labium, near angle of the mouth. Majority of inferior labium supplied via mental artery.
Maxillary Labial	Buccal	Dorsal termination of the enlarged buccal artery. Few anastomoses on lateral nasal surface.	Superior labium. Majority of superior labium supplied via infraorbital artery.

Superficial Temporal	External Carotid	Last retromandibular branch of external carotid. For 2cm, the superficial temporal is essentially a common trunk to transverse facial (anterior continuation), superficial temporal (dorsal continuation), and rostral auricular (caudal continuation) arteries. Superficial temporal crosses zygomatic lateral to the temporomandibular joint and continues superiorly along rostral margin of temporalis muscle. At level of contact between temporal process and zygomatic process of frontal bone arises the lacrimal artery. Divides into lateral superior and lateral inferior palpebral vessels over the postorbital bar. Caudal to the superficial boundary of the orbit, supplies lacrimal gland via eponymous branch.	Lateral face over masseter muscle, lateral orbit, orbicularis oculi muscle, meninges, base of auricle, ossicone.
Transverse Facial	Superficial Temporal	First anterior branch of the superficial temporal. Arises from "trifurcation" with superficial temporal and rostral auricular arteries. Short distribution across lateral face. Deeper branches pierce the superficial surface of posterosuperior masseter muscle, and superficial branches supply the parotid gland.	Masseter, parotid gland.
Lacrimal	Superficial Temporal	Caudal to the superolateral margin of the orbit, the lacrimal artery departs from the superficial temporal. Supplies the lacrimal gland; terminates as lateral palpebral vessels.	Lacrimal gland; palpebrae.
Lateral Superior Palpebral	Superficial Temporal	Originates as termination of lacrimal artery; shares a common trunk with the lateral inferior palpebral at the level of contact between the frontal and zygomatic portions of the lateral orbital bar. Supplies upper palpebrum, lacrimal gland, and lateral orbicularis occuli muscle.	Superior palpebrum, lacrimal gland, and orbicularis oculi muscle.
Lateral Inferior palpebral	Superficial Temporal	Ventral termination of the lacrimal artery, coursing toward and within the inferior palpebrum.	Inferior palpebrum and orbicularis oculi muscle.
Anterior Tympanic	Superficial Temporal	Enters middle ear after branching from the caudal surface of the superficial temporal, before the parent artery reaches the post glenoid foramen.	Tympanum.
Middle Meningeal	Superficial Temporal	Enters temporal meatus via post glenoid foramen. Distributes across caudolateral meninges after exiting the internal opening of the temporal meatus, superior to the petrosal.	Posterolateral meninges.

Cornual (Ossicone)	Superficial Temporal	Dorsal termination of the superficial temporal, and the major artery supplying the developing ossicone. Collateral circulation to the ossicone is by suprarobital arteries. Extensive superficial plexus surrounds ossicone and contributes deeper branchs to internal plexus. As ossicone is developing, there are a number of branches from the cornual artery that contribute to zones of ossification.	Ossicone and frontal/parietal bones at base.
Rostral Auricular	Superficial Temporal	Caudal termination of superficial temporal; arises opposite transverse facial. Shortly after origin, large branch departs and enters temporal meatus (middle meningeal/ temporoparietal meningeal). Superficial branches supply the dorsal portion of the parotid gland and the ventral margin of the auricle. Ramifies caudal portion of temporalis muscle before reaching the auricle. At the auricular base, supplies a deep temporal vessel. Terminates near base of auricle.	Parotid gland, temporalis m., auricular base, auricle, and meninges (indirect).
**Branches of the Maxillary Artery**			
Inferior Alveolar	Maxillary	Originates from ventral surface of the MA between the superficial and deep temporal vessels. Courses laterally to enter the mandibular canal, wherein branches depart for the alveoli of the mandibular dentition. Terminates superficially as the mental artery, and anteriorly by supplying the lower incisors.	Roots of mandibular dentition; continues as mental artery to supply lower lip.
Mental	Inferior Alveolar	Anterior continuation of the inferior alveolar artery. Exits mandibular canal through the mental foramen, before continuing anteriorly to serve as the predominant source of oxygenated blood to lower lip.	Lower lip; partially ramified by sublingual artery.
Caudal Deep Temporal	Maxillary	Direct tributary of the maxillary artery that arises almost immediately dorsal to the inferior alveolar artery. Follows the surface of the temporal bone to supply the temporalis muscle from its deep border. Parent artery contributes laterally-coursing masseteric artery at the level of mandibular incisure. As it passes thru incisure, small branches ramify the anterior temporomandibular joint capsule.	Temporalis and mandibular muscles, temporomandibular joint.
Masseteric	Caudal Deep Temporal	Traverses mandibular incisure (between coronoid and condylar processes) to immediately supply masseter. As it passes through the mandibular incisure, a small branch ramifies the temporomandibular joint.	Masseter muscle, temporomandibular joint.

Ramus Anastomoticus	Maxillary	Arises from the maxillary artery, ascends toward basicranium and enters via the foramen ovale to ramify the carotid rete.	Carotid rete.
Buccal	Maxillary	Originates from ventral surface of maxillary artery in close proxy to the arteria anastomotica. Courses through the pterygopalatine fossa, between the rostral border of the masseter and the maxillary tuberosity. Before reaching the maxillary tuberosity, two arteries depart from the buccal: the rostral deep temporal artery dorsally and the pterygoid branch ventrally. The origin of the pterygoid branch is nearly shared with the maxillary artery. After exiting the fossa between the masseter and maxillary tuberosity, the proximal course of the buccal is typical: a superiorly coursing branch supplies the extraorbital fat and extends to the anterior border of the zygomatic; the rostrally coursing branch supplies the buccinator muscle. Distinctive from the typical giraffid pattern, the distal buccal artery compensates for the reduced facial and transverse facial arteries. It terminates by bifurcating into mandibular and maxillary labial arteries near the 2nd lower molar. The deep surface of the artery is extensively dendritic to buccal glands.	Buccinator muscle, inferior periorbita, buccal glands, mandibular and maxillary labia.
Pterygoid Branches	Maxillary/Buccal	Near contact between buccal and MA, pterygoid departs from ventral surface of buccal. Supplies pterygoid muscles from caudal border. Extensively dendritic within muscular parenchyma. Anterior vessel enters pterygoid bone.	Pterygoid muscles and bone.
Rostral Deep Temporal	Buccal	Small artery originating from the dorsal surface of the buccal artery, proximal to the anterior border of the coronoid process. Lengthy course on deep surface of the temporalis muscle, beginning near the muscle's insertion on the coronoid and throughout the deep temporal fossa.	Temporalis muscle and tendon.
Arteria Anastomotica	Maxillary	Originates near the external ophthalmic artery, within the external, rostral continuation of the carotid rete. Courses caudally and enters the cranium via the foramen ovale. Supplies caudal carotid rete. Contributes hypophyseal vessel.	Carotid rete.
External Ophthalmic	Maxillary/rami anastomotica	The external ophthalmic artery is a large tributary from the dorsal surface of the maxilliary artery. It shares a common trunk with the rami anastomotica, as such its origin is close to the foramen orbitorotundum. The EO courses to the apex of the orbit in close association to the orbitosphenoid. Its tributaries are described below (see: *Arterial Supply to the Eye and Orbit*).	Majority of orbit, eyeball, and periorbita.

Infraorbital	Maxillary	Rostral continuation of the maxillary. From the pterygopalatine fossa, enters the infraorbital canal and distributes to the alveoli of maxillary dentition. Becomes superficial via the infraorbital foramen and contributes to rostrolateral nasal and maxillary labial circulation.	Alveoli of cheek teeth, nasal and maxillary labial soft tissues.
Malar	Infraorbital	At the rostral border of the orbit, the malar departs the infraorbital and courses dorsomedially. Near the inferior margin of the orbit, the medial inferior palpebral departs laterally, along with several ventrally-coursing vessels bound for the orbicularis oculi muscle. As the malar continues dorsally, it subdivides into 3 heavily branching and anastomotic terminals. Caudally, the artery to the angle of the eye extensively perfuses the tissues overlying the dorsal margin of the orbit and the frontal. It anastomoses with the superficial temporal/cornual. The rostral termination is the caudal lateral nasal, which anastomoses with the rostral lateral nasal anteriorly and the dorsal termination of the parent artery dorsally. The dorsal termination is the aptly named dorsal nasal artery.	Lower eyelid and orbicularis oculi muscle, ventral, medial, and dorsomedial margins of the orbit; rostral frontal bone; dorsal and caudal portions of the nasal region; ramifies base of developing ossicone.
Rostral Lateral Nasal	Malar	Dorsal termination of the malar; Courses caudally toward ossicone. Provides collateral circulation to base of ossicone and anastomoses freely with deeper branches of the superficial temporal.	Apex of nasal region; dorsal margin of orbit; base of ossicone; deep rostral border of temporalis m.
Dorsal Nasal	Malar	Malar ascends near ventromedial wall of orbit, turns medial at lacrimal fossa and continues as lateral nasal. Near its origin, several branches pierce the skull to enter the extensive frontal sinuses.	Lateral nasal region; mucosa of frontal sinuses.
Descending Palatine	Maxillary	Rostral termination of the maxillary artery- arises as a bifurcation with the infraorbital. Contributes greater, lesser, and sphenopalatine arteries.	Hard and soft palates, nasal mucosa and maxilloturbinates.
Accessory Lesser Palatine	Maxillary	Small branch distal to terminal bifurcation of maxillary artery; ramifies soft palate.	Rostral soft palate.
Lesser palatine	Descending Palatine	From descending palatine, courses ventrally through lesser palatine foramen/canal. Within muscles and connective tissue of soft palate, courses caudally and toward midline on internal surface of horizontal palatine. At midline, joins contralateral lesser palatine. Continues caudally with major branch reaching the uvula and lateral branches that ramify the pharyngeal vasculature (including direct derivatives of lingual artery).	Soft palate, pharynx.

Greater Palatine	Descending Palatine	Enters caudal palatine foramen and courses through palatine canal. Bifurcates before lower branch exits via rostral palatine foramen. Rostral branch remains internal and supplies floor of nasal cavity. External branch courses in palatal grooves. Near the palatine fissure, joins the contralateral greater palatine and continues rostrally to anterior incisive and keratinous pad.	Nasal floor, hard palate and associated structures, incisive and keratinous pad.
Sphenopalatine	Descending Palatine	Only dorsal branch of descending palatine. Distributes extensively around maxilloturbinates; medial branches supply nasal septum.	Maxilloturbinates; nasal septum.
**Arterial Supply to the Eye and Orbit**			
External Ophthalmic	Maxillary / Rami Anastomotica	The external ophthalmic artery is a large tributary from the dorsal surface of the maxilliary artery. It shares a common trunk with the arteria anastomotica, as such its origin is close to the foramen orbitorotundum. The external ophthalmic courses to the apex of the orbit in close association to the orbitosphenoid. Throughout its course, it supplies the majority of structures associated with the eye and orbit, including the extraocular muscles (not lateral rectus). Receives anastomosis from internal ophthalmic. Ophthalmic retia are restricted to the external ophthalmic artery, and are not confluent with the carotid rete or arteria anastomotica. Branches condense from the ophthalmic rete to serve the globe of the eye. Terminates by bifurcating into external ethmoidal and supraorbital arteries.	Majority of orbit, eyeball, and periorbita.
Internal Ophthalmic	Cerebral Arterial Circle	Courses through the optic foramen and joins the ophthalmic rete. Note: the internal ophthalmic does not directly contact eye or orbital structures.	Connects the cerebral arterial circle to ophthalmic rete; does not directly supply eye.
Central Artery of the Retina	Infraorbital artery	Small artery. From the infraorbital, the central artery of the retina pierces the dura and connective tissue sheath of the optic nerve near the origin of the nerve from the optic chiasm. Courses within nerve tissue to supply retina.	Retina.
Lacrimal	Superficial Temporal	Caudal to the superolateral margin of the orbit, the lacrimal artery departs from the superficial temporal. Supplies the lacrimal gland; terminates as lateral palpebral vessels.	Lacrimal gland; palpebrae.

Lateral Superior Palpebral	Superficial Temporal	Originates as termination of lacrimal artery; shares a common trunk with the lateral inferior palpebral at the level of contact between the frontal and zygomatic portions of the lateral orbital bar. Supplies upper palpebrum lacrimal gland, and lateral orbicularis occuli muscle.	Superior palpebrum, lacrimal gland, and orbicularis oculi muscle.
Lateral Inferior palpebral	Superficial Temporal	Ventral termination of the lacrimal artery, coursing toward and within the inferior palpebrum.	Inferior palpebrum and orbicularis oculi muscle.
