# Supplementary material for: A comparison of postnatal arterial patterns in a growth series of giraffe (Artiodactyla: Giraffa camelopardalis)
Source: PeerJ. 2016 Feb 16;4:e1696. doi: 10.7717/peerj.1696 (PMC4768699; doi:10.7717/peerj.1696)
Supplement: Table S3 [file peerj-04-1696-s003.pdf]

Cranial Arteries of the Stillborn Giraffe

Branches of the External Carotid Artery

Artery	Origin	Course	Distribution
External Carotid	Common Carotid Artery @ Occipital	Begins at occipital artery. There is no remnant of the internal carotid artery to easily demarcate division of the common carotid into the external carotid.	Supplies entirety of cranium with oxygenated blood. Continues rostrally as maxillary artery near departure of inferior alveolar artery.
Occipital	External Carotid	First branch of the external carotid; departs from dorsal surface near tip of jugular process. Ascends toward condylar foramen/occipital bone. Before condylar foramen, contributes condylar artery. Follows caudal surface of temporal crest, superficial to the weakly-developed bone. Caudal meningeal branch enters mastoid foramen within mastoid fossa.	Gives off few muscular branches. Additional distribution is to the caudal meninges.
Condylar	Occipital	Short extracranial course to reach condylar canal. On internal surface of occipital condyle, a caudal meningeal artery departs. Presumably anastomoses with vertebral artery; however, vertebral arteries are not intact. Does not contact cerebral arterial circle.	Distributes only to caudal meninges and vertebral artery.
Caudal Auricular	External Carotid	3cm distal to occipital artery; begins rostral to jugular process, courses obliquely across and superficial to jugular and mastoid, does not contact or leave groove on mastoid. Bifurcates into caudal and medial auricular arteries posteriorly. Considerable anastomosis with rostral auricular.	Majority of auricle supplied through caudal auricular
Stylomastoid	*Indeterminable*		
Deep Auricular	Caudal Auricular	Dorsal termination of caudal auricular; after several centimeters, penetrates caudal temporalis muscle.	Auricular base, temporalis muscle.
Lingual	External Carotid	Largest rostroventral branch of ECA. Does not arise from shared trunk with facial. Due to tongue resection, approximately 1cm of the lingual artery remains.	Presumably as in adult and juvenile giraffes.
Deep Lingual	*Necropsied*		
Sublingual			

Facial	External Carotid	Originates from rostroventral wall of external carotid, a short distance from the stalk of the lingual artery. Courses rostrally, becoming superficial within a concavity between the angle and body of the mandible. Presumably follows pattern of adult giraffe. Bifurcates superficial to the mandibular molar row into rostral and dorsal coursing branches. Terminations are not perfused.	Difficult to assess dorsal distribution given degree of necropsy (entirety of buccal region was transected). Course is obscured anterior to the mandibular molar row; however, bifurcation follows pattern of adult giraffe.
Mandibular Labial	Facial	Originates from bifurcation of facial artery; incompletely perfused.	Incompletely perfused. Distribution presumed similar to adult giraffe (inferior labium).
Maxillary Labial	Facial	Originates from bifurcation of facial artery; incompletely perfused.	Incompletely perfused. Distribution presumed similar to adult giraffe (superior labium). Superior labium receives most arterial blood from infraorbital artery.
Superficial Temporal	External Carotid	Last retromandibular branch of the external carotid. From proximal to distal, parent artery to the transverse facial (first rostral branch), rostral auricular (first caudal branch), lacrimal (second rostral branch), and cornual artery (dorsal continuation).	Lateral face over masseter muscle, lateral orbit, orbicularis oculi muscle, meninges, base of auricle, ossicone.
Transverse Facial	Superficial Temporal	First rostral branch of the superficial temporal. Arises several centimeters below the rostral auricular. Short distribution across lateral face. Deeper branches pierce the superficial surface of caudodorsal masseter muscle, superficial branches supply the parotid gland.	Masseter muscle, parotid gland.
Lacrimal	Superficial Temporal	Caudal to the dorsolateral margin of the orbit, the lacrimal artery departs from the superficial temporal. Supplies the lacrimal gland, superior and inferior lateral palpebrae and orbicularis oculi muscles.	Lacrimal gland, palpebrae, orbicularis oculi muscles.
Lateral Superior Palpebral	Superficial Temporal	Originates as termination of lacrimal artery; shares a common trunk with the lateral inferior palpebral at the level of contact between the frontal and zygomatic portions of the lateral orbital bar. Supplies upper palpebrum, lacrimal gland, and lateral orbicularis occuli muscle.	Superior palpebrum, lacrimal gland, and orbicularis oculi muscle.
Lateral Inferior palpebral	Superficial Temporal	Ventral termination of the lacrimal artery, coursing toward and within the inferior palpebrum.	Inferior palpebrum and orbicularis oculi muscle.
Anterior Tympanic	*Indeterminable*		

Middle Meningeal	Superficial Temporal	Enters large, open temporal meatus via post glenoid foramen. Distributes across caudolateral meninges after exiting the internal opening of the temporal meatus, dorsal to the petrosal.	Caudolateral meninges.
Cornual (Ossicone)	Superficial Temporal	Dorsal termination of the superficial temporal artery, and major supplicant of the incipient ossicone. Extensive plexus underlies lens of connective tissue.	Ossicone and frontal/parietal bones at base.
Rostral Auricular	Superficial Temporal	Major caudal branch of the superficial temporal, originating ventral to the zygoma. Shortly after origin, large branch departs and enters temporal meatus (middle meningeal artery). Superficial branches supply the dorsal portion of the parotid gland and the vengral margin of the auricle. Ramifies caudal portion of temporalis muscle before reaching the auricle. Terminates near base of auricle	Parotid gland, temporalis muscle, auricular base, auricle, meninges (indirect).
**Branches of the Maxillary Artery**			
Inferior Alveolar	Maxillary	Originates from ventral surface of the MA between the superficial and deep temporal vessels. Courses laterally to enter the mandibular canal, wherein branches depart for the alveoli of the mandibular dentition. Terminates superficially as the mental artery, and rostrally by supplying the lower incisors.	Roots of mandibular dentition; continues as mental artery to supply inferior labium.
Mental	Inferior Alveolar	Incompletely perfused, but presumed to be the predominant source of oxygenated blood to lower lip.	Inferior labium.
Caudal Deep Temporal	Maxillary	Direct tributary of the maxillary artery that arises almost immediately dorsal to the inferior alveolar. Incompletely perfused; course presumed similar as juvnile and adult giraffes.	Temporalis and masseter muscles, temporomandibular joint.
Masseteric	Caudal Deep Temporal	Traverses mandibular incisure (between coronoid and condylar processes) to immediately supply masseter.	Masseter muscle.
Ramus Anastomoticus	Maxillary	Arises from the maxillary artery, ascends toward basicranium and enters via the foramen ovale to ramify the carotid rete.	Carotid rete.

Buccal	Maxillary	Stems from ventral surface of maxillary artery in close proxy to the arteria anastomotica/external ophthalmic trunk. Courses through the pterygopalatine fossa, between the rostral border of the masseter and the maxillary tuberosity. Before reaching the maxillary tuberosity, two arteries depart from the buccal: the rostral deep temporal artery dorsally and the pterygoid branches ventrally. After exiting the fossa between the masseter and maxillary tuberosity, the proximal course is indistinguishable due to incomplete perfusion.	Lateral face, buccinator muscle.
Pterygoid Branches	Maxillary/Buccal	Near contact between buccal and maxillary artery, pterygoid branches depart from ventral surface of buccal. Supplies pterygoid muscles. Extensively dendritic within muscular parenchyma.	Pterygoid muscles.
Rostral Deep Temporal	Buccal	Small artery originating from dorsal surface of buccal a. proximal to the coronoid process/anterior border of mandibular ramus. Short course on deep surface of the temporalis m. near its insertion on the coronoid.	Distal tendon and muscle of temporalis.
Arteria Anastomotica	Maxillary	Originates near the external ophthalmic artery, within the external rostral continuation of the carotid rete. Courses caudally and enters the cranium via the foramen ovale. Supplies caudal carotid rete. Contributes hypophyseal vessel.	Carotid rete.
External Ophthalmic	Maxillary/rami anastomotica	The external ophthalmic artery is a large tributary from the superior surface of the maxilliary artery. It shares a common trunk with the rami anastomotica, as such its origin is close to the foramen orbitorotundum. The external ophthalmic courses to the apex of the orbit in close association to the orbitosphenoid. Its tributaries are described below (see: Arterial Supply to the Eye and Orbit).	Majority of orbit, eyeball, and periorbita.
Infraorbital	Maxillary	Rostral continuation of maxillary artery. From pterygopalatine fossa, enters infraorbital canal; distributes to alveoli of maxillary dentition; exits via infraorbital foramen and contributes to rostrolateral nasal and maxillary labial circulation.	Alveoli of cheek teeth, nasal and maxillary labial soft tissues.

Malar	Infraorbital	At the rostral border of the orbit, the malar departs the infraorbital and courses dorsomedially. Near the ventral margin of the orbit, the medial inferior palpebral departs laterally, along with several ventrally-coursing vessels bound for the orbicularis oculi muscle. As the malar continues superiorly, it departs into 3 heavily branching and anastomotic terminals. Caudally, the artery to the angle of the eye extensively perfuses the tissues overlying the dorsal margin of the orbit and the frontal. It anastomoses with the superficial temporal/cornual. The rostral termination is the caudal lateral nasal, which anastomoses with the rostral lateral nasal anteriorly and the superior termination of the parent artery dorsally. The superior termination is the aptly named dorsal nasal artery.	Lower eyelid and orbicularis oculi m., ventral, medial, and dorsomedial margins of the orbit; rostral frontal bone; dorsal and caudal portions of the nasal region.
Rostral Lateral Nasal	Malar	Dorsal termination of the malar; Courses caudally toward ossicone. Provides collateral circulation to base of ossicone and anastomoses freely with deeper branches of the superficial temporal.	Apex of nasal region; dorsal margin of orbit; base of ossicone; deep rostral border of temporalis muscle.
Dorsal Nasal	Malar	Malar ascends near ventromedial wall of orbit, turns medial at lacrimal fossa and continues as lateral nasal. Near its origin, several branches pierce the skull to enter the extensive frontal sinuses.	Lateral nasal region; mucosa of frontal sinuses.
Descending Palatine	Maxillary	Rostral termination of maxillary artery / bifurcation with infraorbital. Contributes greater, lesser, and sphenopalatine arteries.	Hard and soft palates, nasal mucosa, and maxilloturbinals.
Accessory Lesser Palatine	*Indeterminable*		
Lesser palatine	Descending palatine	From descending palatine, courses ventrally through lesser palatine foramen/canal. Within muscles and connective tissue of soft palate, courses caudally and toward midline on internal surface of horizontal palatine. At midline, joins contralateral lesser palatine. Continues caudally with major branch reaching the uvula and lateral branches that ramify the pharyngeal vasculature (including direct derivatives of lingual artery).	Soft palate, pharynx.

Greater Palatine	Descending palatine	Enters caudal palatine foramen and courses through palatine canal. Bifurcates before lower branch exits via rostral palatine foramen. Rostral branch remains internal and supplies floor of nasal cavity. External branch courses in palatal grooves. Near the palatine fissure, joins the contralateral greater palatine and continues rostrally to anterior incisive and keratinous pad.	Nasal floor, hard palate and associated structures, incisive and keratinous pad.
Sphenopalatine	Descending palatine	Only dorsal branch of descending palatine. Distributes extensively around maxilloturbinals; medial branches supply nasal septum.	Maxilloturbinals; nasal septum.
**Arterial Supply to the Eye and Orbit**			
External Ophthalmic	Maxillary / Rami Anastomotica	The external ophthalmic artery is a large tributary from the dorsal surface of the maxilliary artery. It shares a common trunk with the arteria anastomotica, as such its origin is close to the foramen orbitorotundum. The external ophthalmic courses to the apex of the orbit in close association to the orbitosphenoid. Throughout its course, it supplies the majority of structures associated with the eye and orbit, including the extraocular muscles (not lateral rectus). Receives anastomosis from internal ophthalmic. Ophthalmic retia are well developed in the stillborn specimen. Branches condense from the ophthalmic rete to serve the globe of the eye. Terminates by bifurcating into external ethmoidal and supraorbital arteries.	Majority of orbit, eyeball, and periorbita.
Internal Ophthalmic	*Indeterminable*		
Central Artery of the Retina			
Lacrimal	Superficial Temporal	Caudal to the dorsolateral margin of the orbit, the lacrimal artery departs from the superficial temporal. Supplies the lacrimal gland; terminates as lateral palpebral vessels.	Lacrimal gland; palpebrae.
Lateral Superior Palpebral	Superficial Temporal	Originates as termination of lacrimal artery; shares a common trunk with the lateral inferior palpebral at the level of contact between the frontal and zygomatic portions of the lateral orbital bar. Supplies upper palpebrum lacrimal gland, and lateral orbicularis occuli muscle.	Superior palpebrum, lacrimal gland, and orbicularis oculi muscle.
Lateral Inferior palpebral	Superficial Temporal	Ventral termination of the lacrimal artery, coursing toward and within the inferior palpebrum.	Inferior palpebrum and orbicularis oculi muscle.
