# Supplementary figures and images for: A comparison of postnatal arterial patterns in a growth series of giraffe (Artiodactyla: Giraffa camelopardalis)
Source: PeerJ. 2016 Feb 16;4:e1696. doi: 10.7717/peerj.1696 (PMC4768699; doi:10.7717/peerj.1696)

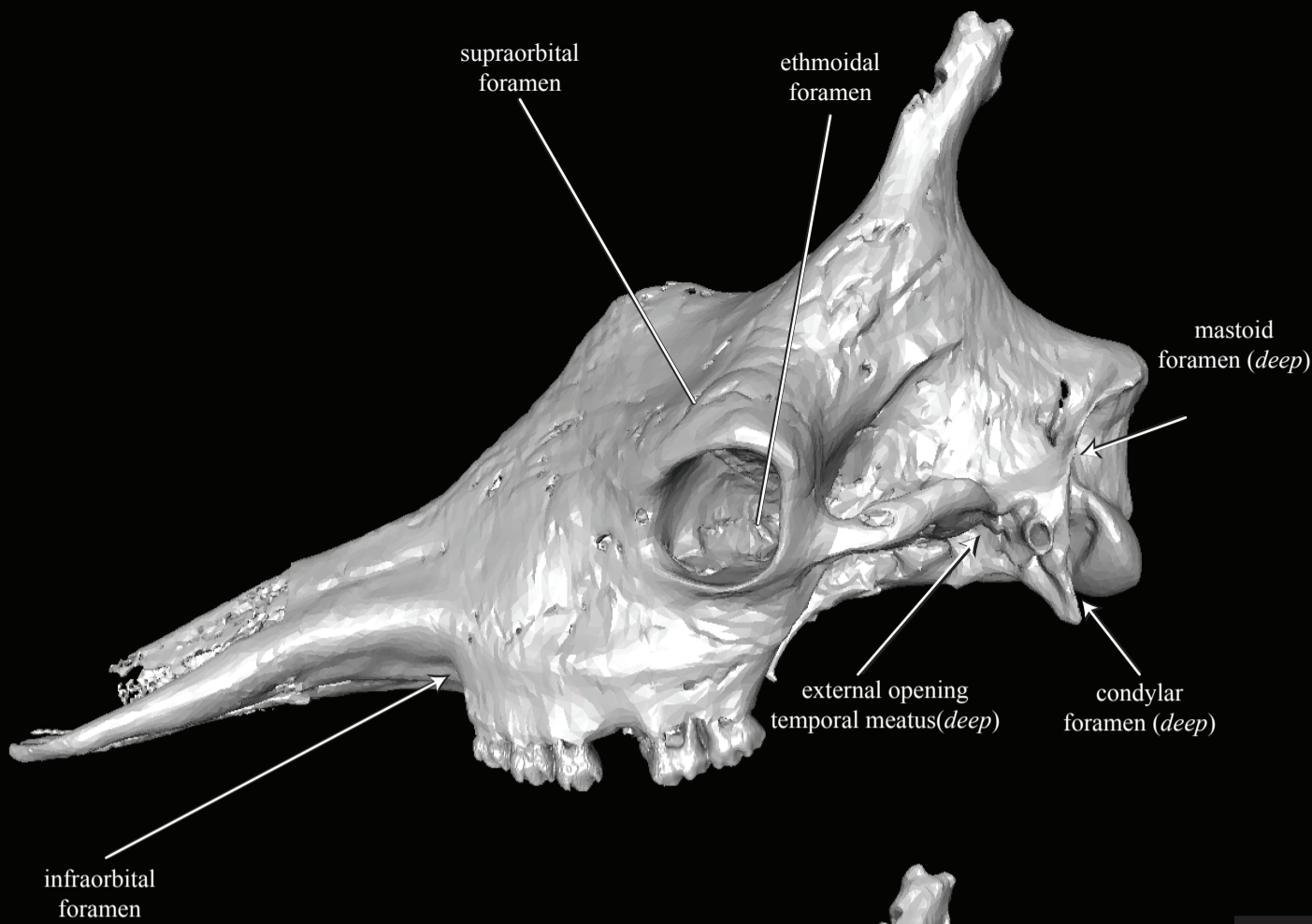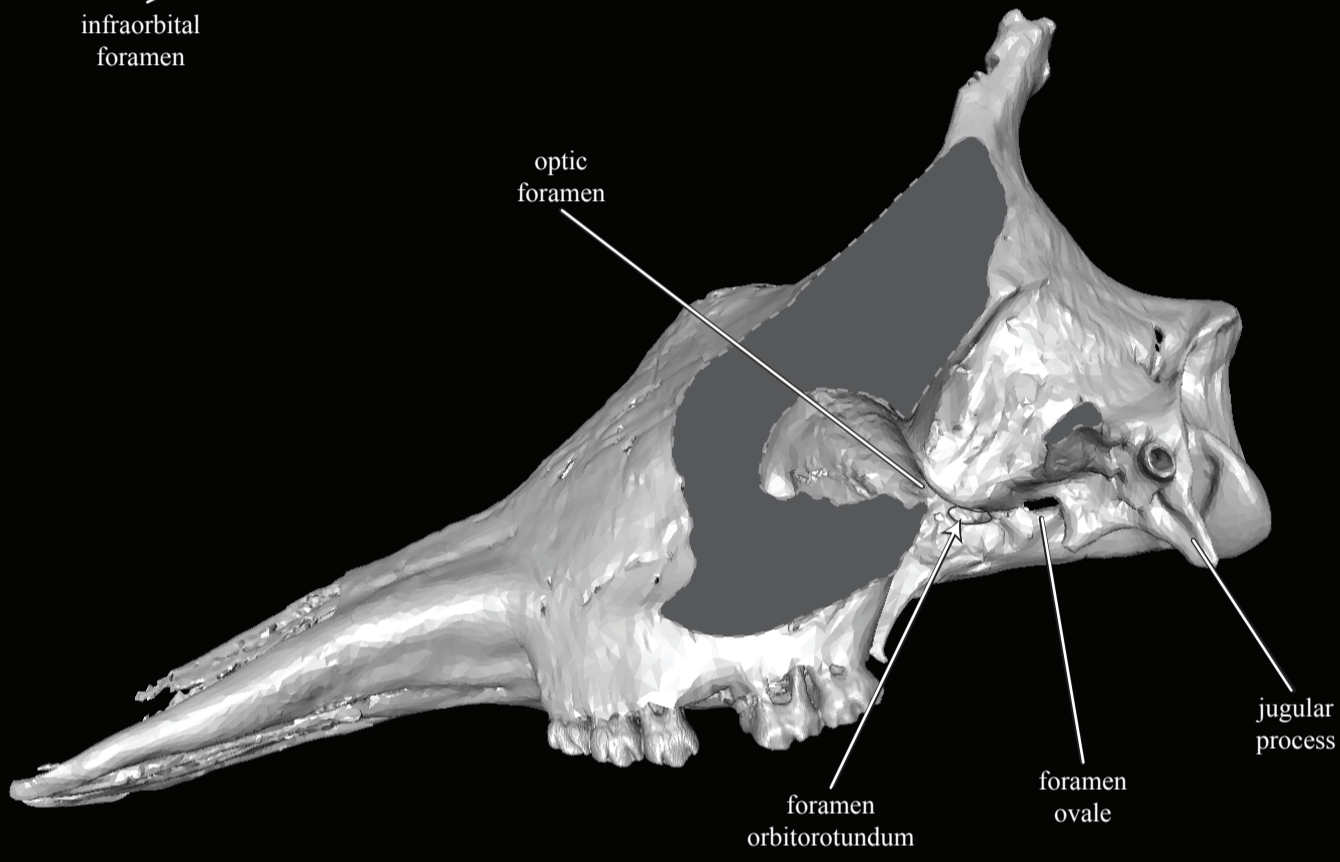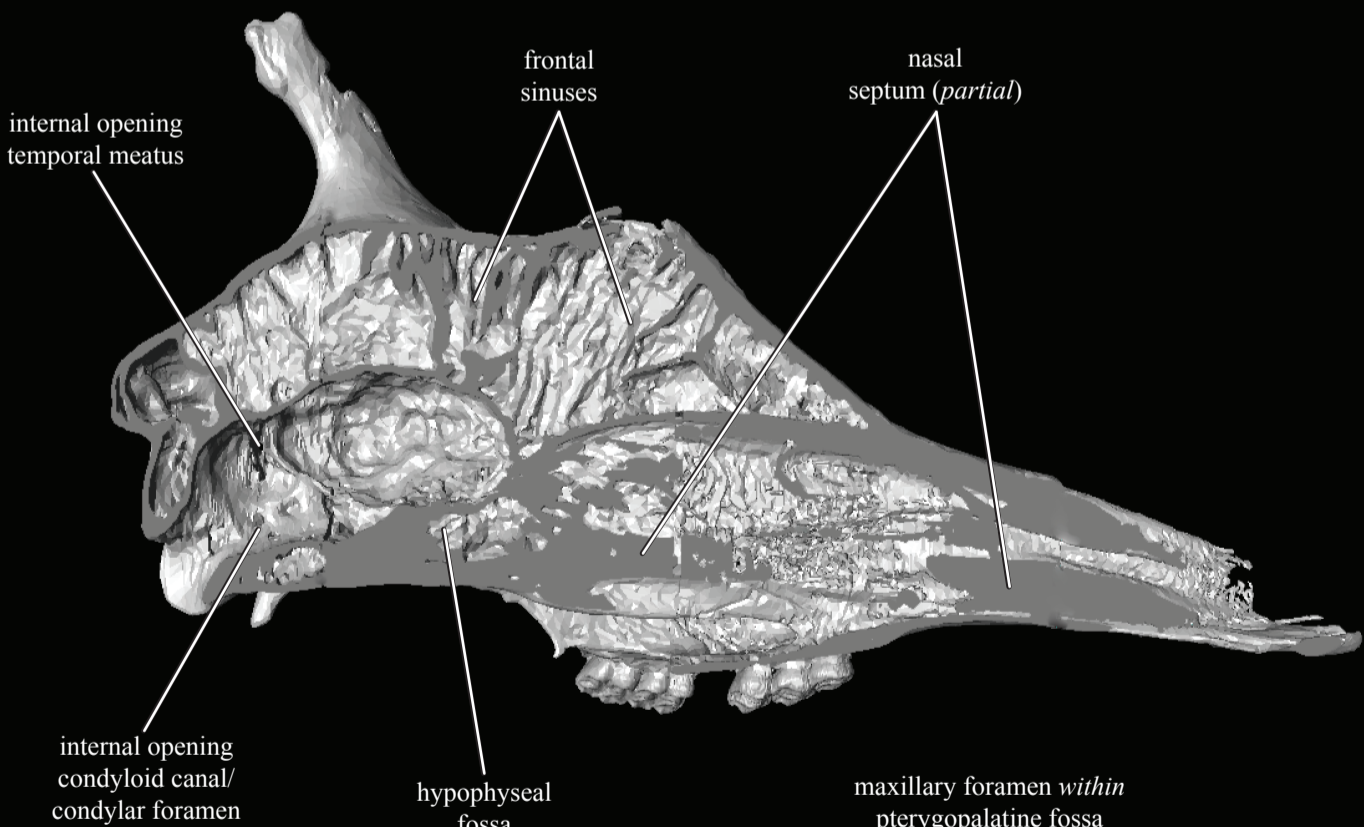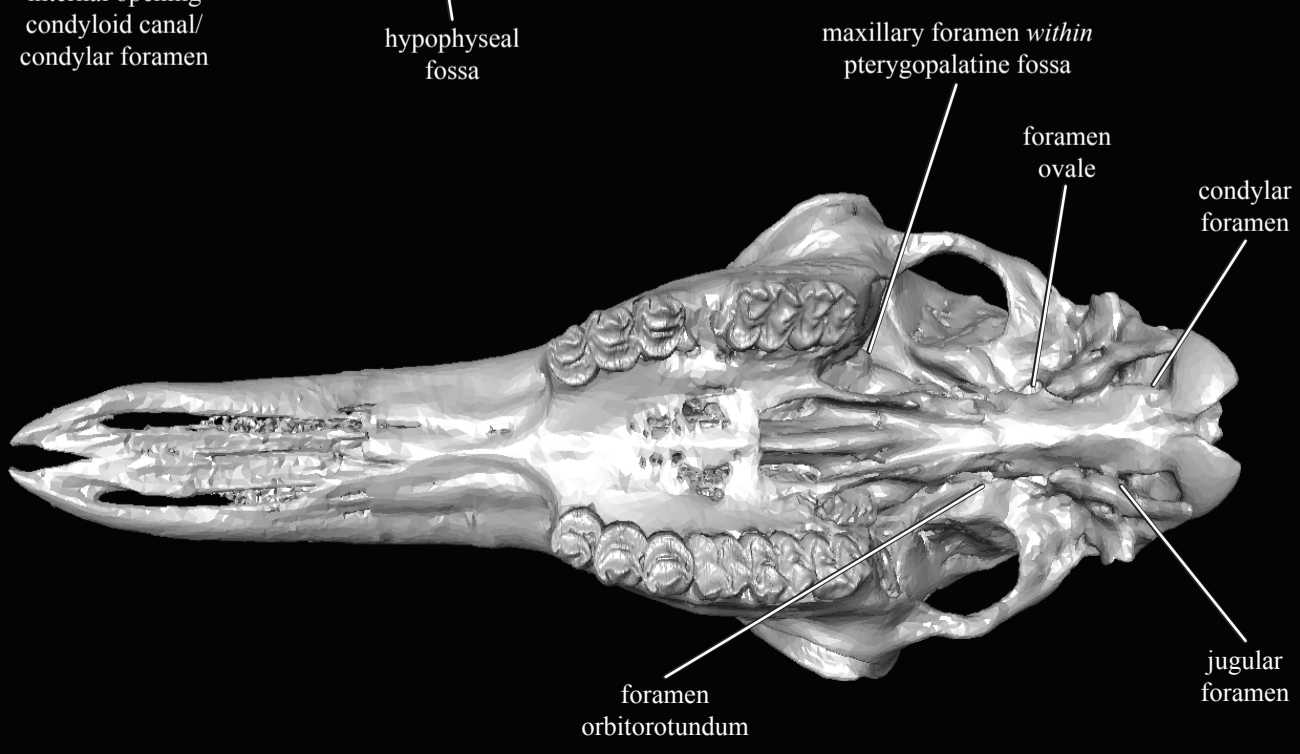

Supplement: Figure S1 [file peerj-04-1696-s004.pdf]
